# c-Myc transactivates *CFL1* to induce senescence-like phenotype and potentiate the bystander effects for the migration and proliferation in lung cancer cells

**DOI:** 10.1038/s41420-026-03065-3

**Published:** 2026-03-26

**Authors:** Yen-Ting Chou, Jyh-Der Leu, Wan-Yu Yang, Chien-Hsiu Li, Min-Ying Lin, Chia-Wei Kao, Yu-Chan Chang, Michael Hsiao, Yi-Jang Lee

**Affiliations:** 1 Department of Biomedical Imaging and Radiological Sciences, National Yang Ming Chiao Tung University, Taipei Branch, Taipei, 112 Taiwan; 2 Division of Radiation Oncology, Taipei City Hospital Ren Ai Branch, Taipei, Taiwan; 3 Department of Urology, Shuang Ho Hospital, Taipei Medical University, New Taipei City, Taiwan; 4 Genomics Research Center. Academia Sinica, Taipei, 11529, Taiwan; 5 Department and Graduate Institute of Veterinary Medicine, National Taiwan University, Taipei, 112 Taiwan; 6 Cancer and Immunology Research Center, National Yang Ming Chiao Tung University, Taipei Branch, Taipei, 112 Taiwan

**Keywords:** Senescence, Oncogenes

## Abstract

Oncogene-induced senescence (OIS) is regarded a tumor suppressive mechanism in normal cells. Accumulated evidences, however, demonstrate that OIS would play a role in cancer promotion through the secretion of senescence associated secretory phenotypes (SASP). The underlying mechanisms remain to be addressed. In this study, we found that c-Myc oncogene could induce senescence in human diploid lung fibroblasts and non-small cell lung cancer cells (NSCLC) without concomitant emergence of apoptosis. c-Myc-induced senescence (cMIS) caused morphological enlargement, increased F-actin and nuclear G-actin that generally detected in senescent cells. These events were found to be associated with increased expression of cofilin-1, an actin-binding protein required for actin dynamics. Transfection of c-Myc could induce cofilin-1, but transfection of truncated Myc-Nick mutant and inhibition of c-Myc reduced cofilin-1 expression. Additionally, knockdown of cofilin-1 could suppress cMIS. The chromatin immunoprecipitation-quantitative polymerase chain reaction (ChIP-qPCR) assay showed that the endogenous c-Myc mainly bound to two out of three predicted E-boxes located in middle and proximity to the transcription initiation site of the *CFL1* promoter. Interestingly, ectopic expression of c-Myc bound to all E-boxes, especially the distal one. Furthermore, the conditioned medium (CM) collected from cells with cMIS could enhance the proliferation and migration of other NSCLC cells, whereas that obtained from cofilin-1 silencing cells with forced expression of c-Myc diminished these capacities. The c-Myc transactivated cofilin-1 could also be triggered by H_2_O_2_ through the middle E-box. Surprisingly, a physical interaction between c-Myc and cofilin-1 was detected, and H_2_O_2_ increased this effect. Clinically, high expression of both *c-Myc* and *CFL1* genes correlated to worse survival rates among NSCLC patients, especially those with the adenocarcinoma subtype. Taken together, the c-Myc-cofilin-1 regulatory axis would explain the mechanism of OIS promoted cancer progression, and it may be a potent target for design of treatments.

## Introduction

Oncogene activation in normal eukaryotic cells is critical for tumorigenesis. However, oncogenes not only promote the rapid progression through the cell cycle but also foster senescence, so called oncogene-induced senescence (OIS). Ectopic expression of mutant form of *Ras* proto-oncogene, known as *Hras*^*G12V*^ is the first example of OIS observed in human lung IMR90 cells [1]. RAS/MAPK and PI3K/AKT pathways are known as key mechanisms of OIS [2]. The RAS/MAPK pathway induces the DNA damage response (DDR) followed by activation of p53/p21^CIP1^ and p16^INK^/Rb networks [3, 4]. On the contrary, the PI3K/AKT pathway triggered OIS is independent of DDR but still requires functional p53/p21^CIP1^ signaling axis via mTORC1 [5]. Despite being ostensibly regarded a tumor suppressor mechanism, OIS belongs to a telomere erosion-independent form of senescence and may serve as an unstable barrier against tumorigenesis [6, 7]. On the other hand, the uncontrolled release of senescence-associated secretory phenotypes (SASP), which act in a paracrine manner to promote cancer progression, represents the dark side of OIS [8]. However, the detailed mechanisms remain to be addressed.

*c-Myc* is another important oncogene that encodes c-Myc oncoprotein known as a multifunctional transcription factor. c-Myc and Max form heterodimers as master transcriptional regulators binding to consensus DNA sequences (CANNTG) named Enhancer-box (E-box) located in the promoters affected genes [9]. c-Myc transactivates multiple genes that control cell cycle, DNA repair, apoptosis, metabolism, self-renewal, ribosomal biogenesis, cell differentiation, tumor microenvironment, and even immune response [10–18]. Over-expression of *c-Myc* is the primary oncogenic action contributing to the cause of 70% of human cancers [19]. However, c-Myc also plays a role in OIS through induction of DDR and generation of reactive oxygen species (ROS) [20]. For instance, c-Myc-induced senescence (cMIS) can be prevented by Werner syndrome protein (WRN), a DNA helicase involved in 12DNA repair mechanism [21]. Additionally, cMIS has been reported to be suppressed by cyclin-dependent kinase 2 (CDK2) [22, 23]. Activation of c-Myc also transcriptionally induction of p14ARF tumor suppressor and ubiquitin-specific protease 10 (USP10) for stabilizing p14ARF to promote cell senescence [4, 24]. Although cMIS may also function as one of the tumor suppressive mechanisms, whether c-Myc mediated OIS that potentially promotes malignant progression, as mentioned above is still little investigated.

The actin depolymerizing factor (ADF)/cofilin family includes a non-muscle isoform named cofilin-1 (~19kD) that is ubiquitously expressed in different organs of mammalian cells [25]. Cofilin-1 encoded by *CFL1* gene is responsible for accelerating the actin dynamics in vitro and in vivo, and is essential for organization of cellular morphology, motility, and polarity [26, 27]. As the morphological change is an obvious characteristic of cell senescence, up-regulation of cofilin-1 via reduced protein degradation rate has been reported to be detected in replicative senescence in vitro and in vivo [28]. On the other hand, increased expression of cofilin-1 is associated with poor prognosis and low survival rates in various human cancers [29–38]. However, it remains unclear whether *CFL1* gene can be transactivated to influence the development of cancer cells.

In this study, we have demonstrated that ectopic expression of c-Myc induced G0/G1 arrest and senescence, but not apoptosis in human diploid fibroblasts and non-small cell lung cancer (NSCLC) cells with or without p53 expression. Over-expression of c-Myc led to reorganization of actin architectures accompanied by up-regulation of cofilin-1 but not other examined actin-associated proteins. The nuclear entry of over-expressed c-Myc is required for induction of cofilin-1 and cell senescence, and it can bind to three E-boxes located in the promoter of *CFL1* gene with different capacities. Cofilin-1 was not only important for c-Myc induced senescence but also the bystander effect that enhanced the migration and proliferation of cancer cells without the transduction of c-Myc. Clinically, high expression of *c-Myc* and *CFL* mainly contribute to the poor survival of lung adenocarcinoma but not that of lung squamous cell carcinoma. Together, the c-Myc/cofilin-1 regulatory axis may shed light on the adverse effect of c-Myc induced senescence in promoting cancer progression.

## Results

### Over-expression of c-Myc increased G1 phase and induced cell senescence

**Fig. 1:**
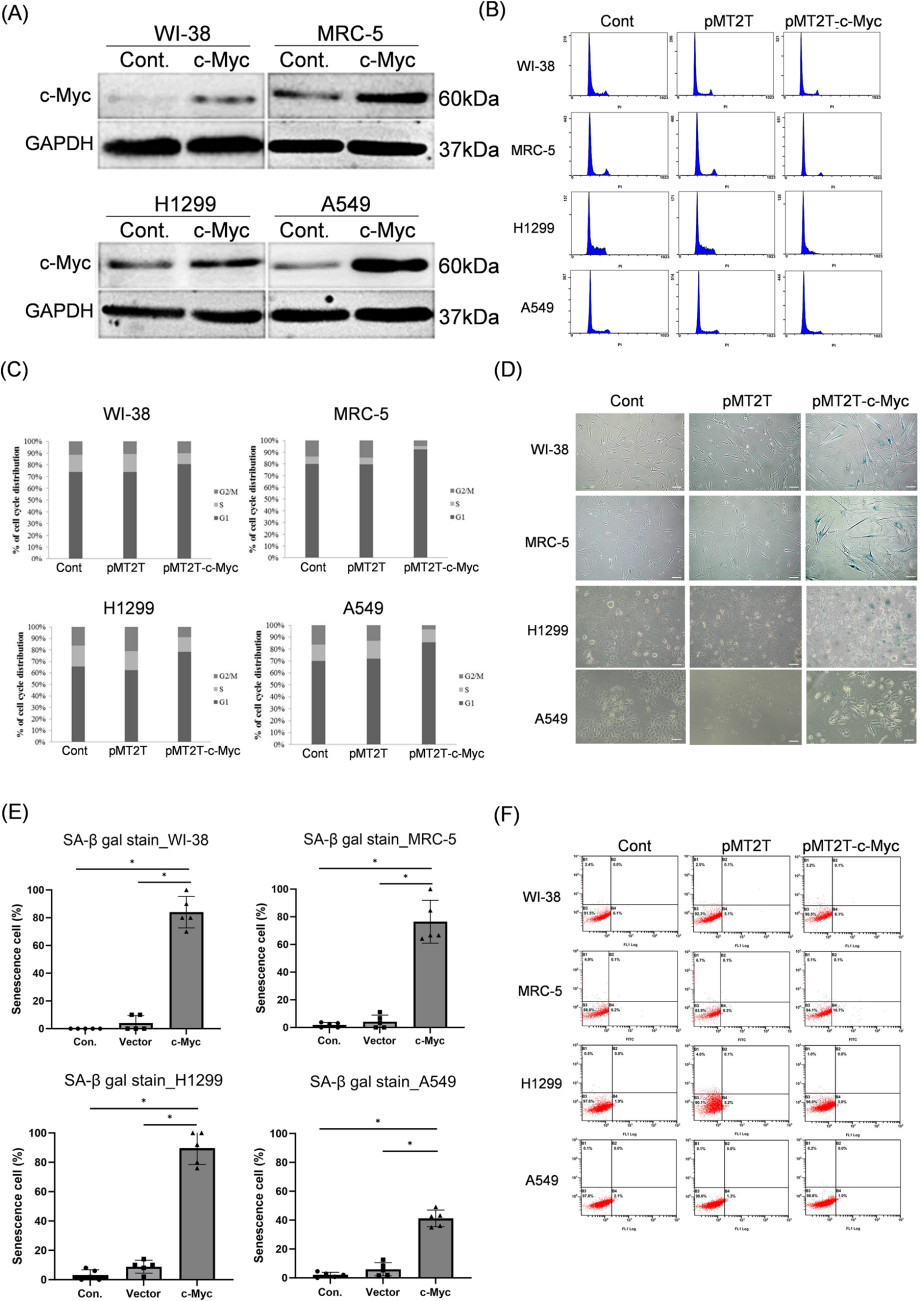
Ectopic expression of c-Myc induced cellular senescence. **A** Western blot analysis for detection of c-Myc transduced into human diploid lung cells and human lung adenocarcinoma cells; **B** DNA histogram for determination of cell cycle distribution after ectopic expression of c-Myc in cells; **C** measurement of percentage of cell cycle phases; **D** SA-β-gal staining of cells transduced with c-Myc expressive plasmid; **E** quantification and comparison of SA-β-gal cells transduced with c-Myc expressive plasmid. Scale bar: 100 μm; **F** detection of apoptotic cells after transduction of c-Myc using the Annexin V-FITC and PI double staining assay. *: *p* < 0.05.

To investigate the effect of c-Myc on cell cycle and viability, we transduced c-Myc gene in different lung cell types including WI-38 cells, MRC-5 cells, A549 cells, and H1299 cells. The transfection efficiency of each cell type reached 90% using the pEGFP-N1 plasmid and visualized under the fluorescence microscope (Supplementary Fig. 1). The patterns of c-Myc over-expression in these cell types were confirmed using the Western blot analysis (Fig. 1A). Over-expression of c-Myc induced G1 phase arrest in all of these cells after 48 h of transfection (Fig. 1B), and more than 80% of cells arrested in G1 phase could be detected (Fig. 1C). The cells were further examined by the SA-β-gal staining analysis that demonstrated c-Myc could induce SA-β-gal expression accordingly (Fig. 1D). The amount the stained cells in control cells (untransfected and vector transfected) and c-Myc transduced cells was also quantified and compared (Fig. 1E). Nevertheless, over-expression of c-Myc did not induce apoptosis by visualizing the analysis of PI-Annexin V staining (Fig. 1F). Nevertheless, a longer time of c-Myc transfection could maintain the G1 phase arrest in A549 cells but not in WI-38 cells and H1299 cells (Supplementary Fig. 2).

### Up-regulation of cofilin-1 is associated with c-Myc induced senescent morphology

**Fig. 2:**
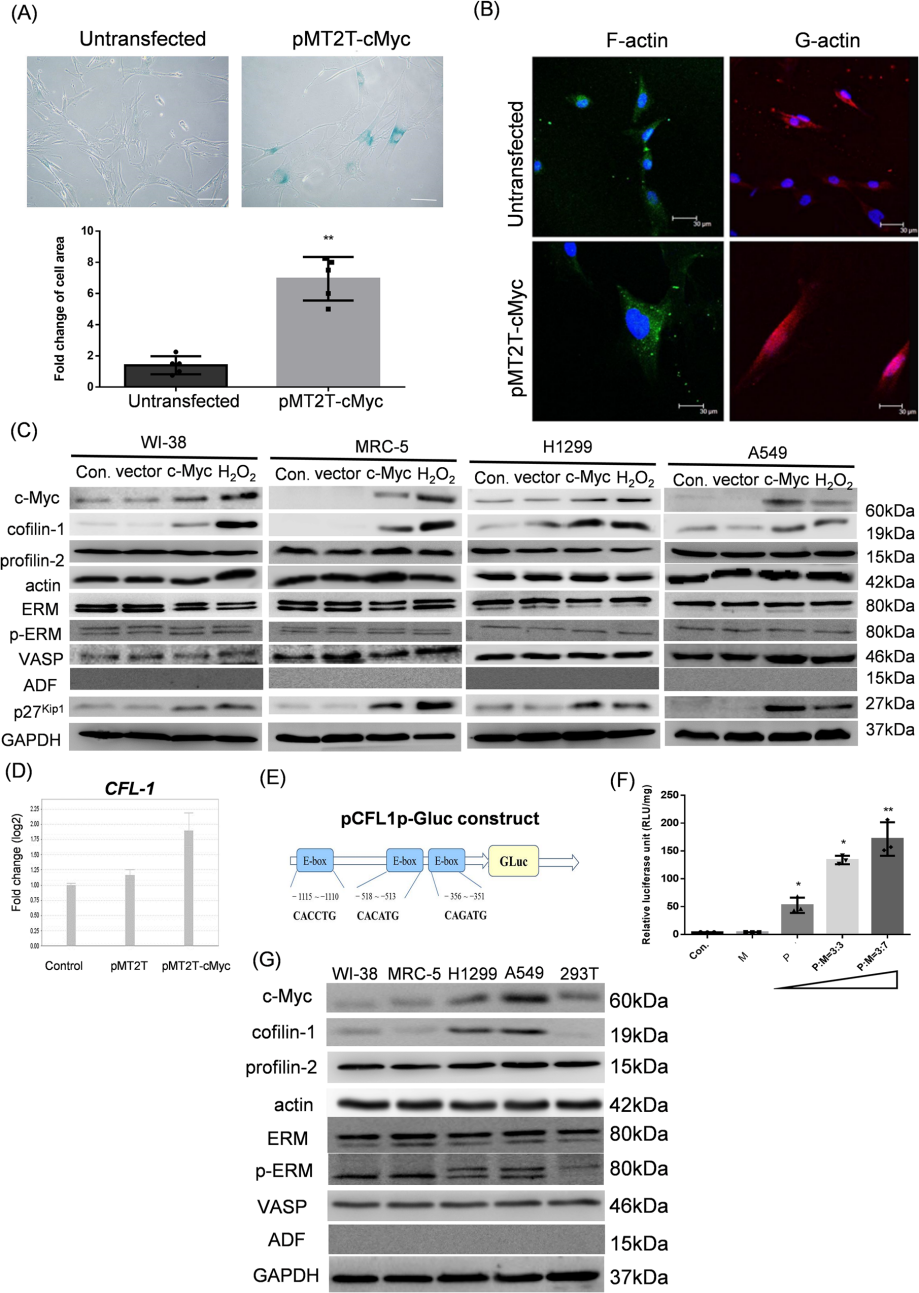
Ectopic expression of c-Myc-induced senescence was associated with up-regulation of cofilin-1. **A** Measurement of morphological change of MRC-5 cells transduced with c-Myc; **B** visualization of F-actin and G-actin using fluorescence microscopy with fluorescein conjugated phalloidin and DNase I, respectively; **C** use of actin analysis for detection of various actin-associated proteins using Western blot analysis; **D** detection of cofilin-1 mRNA using RT-qPCR; **E** a diagram of CFL1 promoter fused to GLuc reporter gene; **F** measurement of CFL1 promoter activity using the in vitro luciferase reporter gene assay. P: pCFL1p-GLuc; M: pMT2T-c-Myc; **G** comparison of the basal protein levels of c-Myc and actin-associated proteins in different cell lines. *: *p* < 0.05; **: *p* < 0.01.

Transduction of c-Myc leads to an enlargement of cell shape visualized by the bright microscope. We measured the cell area and showed a 3-fold increase in the size of MRC-5 cells (Fig. 2A). Staining of F-actin and G-actin showed that increases of cytoplasmic F-actin and cellular G-actin in c-Myc transfected cells, suggesting that the actin turnover was affected (Fig. 2B). We next examined if over-expression of c-Myc would regulate the expression of actin-associated proteins (AAPs). The results showed that transduction of c-Myc to different cell types could all induce CFL-1, but not other AAPs examined, including profiling-2. ERM, -ERM, VASP, and ADF/destrin (Fig. 2C). We also found that H_2_O_2_ could concomitantly induce c-Myc and CFL-1, and the cell cycle inhibitor p27^Kip1^ could be induced by c-Myc and H_2_O_2_ (Fig. 2C). The qPCR analysis demonstrated that transduction of c-Myc could induce the transcription of *CFL-1* gene (Fig. 2D). Using the “Transcriptional Regulatory Element Database (TRED)” online tool that collects mammalian *cis*- and *trans*-regulatory elements together with experimental evidence [39], three separate E-box elements have been predicted on the 1 kb DNA sequence upstream of transcription initiation site (Fig. 2E). c-Myc transcription factor is known to bind to E-box for gene expression [40, 41], and we used the pMyc-TA-Luc reporter construct harboring 6 tandem repeats of E-box to demonstrate that the luciferase reporter gene expression could be driven by co-transfection of c-Myc expressive plasmids (Supplementary fig. 3). To investigate if these E-box elements are important for cofilin-1 gene expression, a Guassia luciferase (Gluc) reporter gene fused to the 1 kb cofilin-1 gene promoter was co-transfected with c-Myc expressing plasmids. The results showed an incremental expression of Gluc activity by increase of c-Myc expressing plasmids (Fig. 2F). Furthermore, we compared the endogenous expressive levels of c-Myc and AAPs in these cell lines, and the results showed that only c-Myc and CFL-1 were co-upregulated in lung cancer A549 cells and H1299 cells, but not in the diploid human lung fibroblasts and human embryonic kidney 293T cells (Fig. 2G). These results suggest that a regulatory axis should exist between c-Myc and *CFL-1* genes.

### Nuclear entry of c-Myc for DNA binding is required for induction of cofilin-1 and cell senescence

**Fig. 3:**
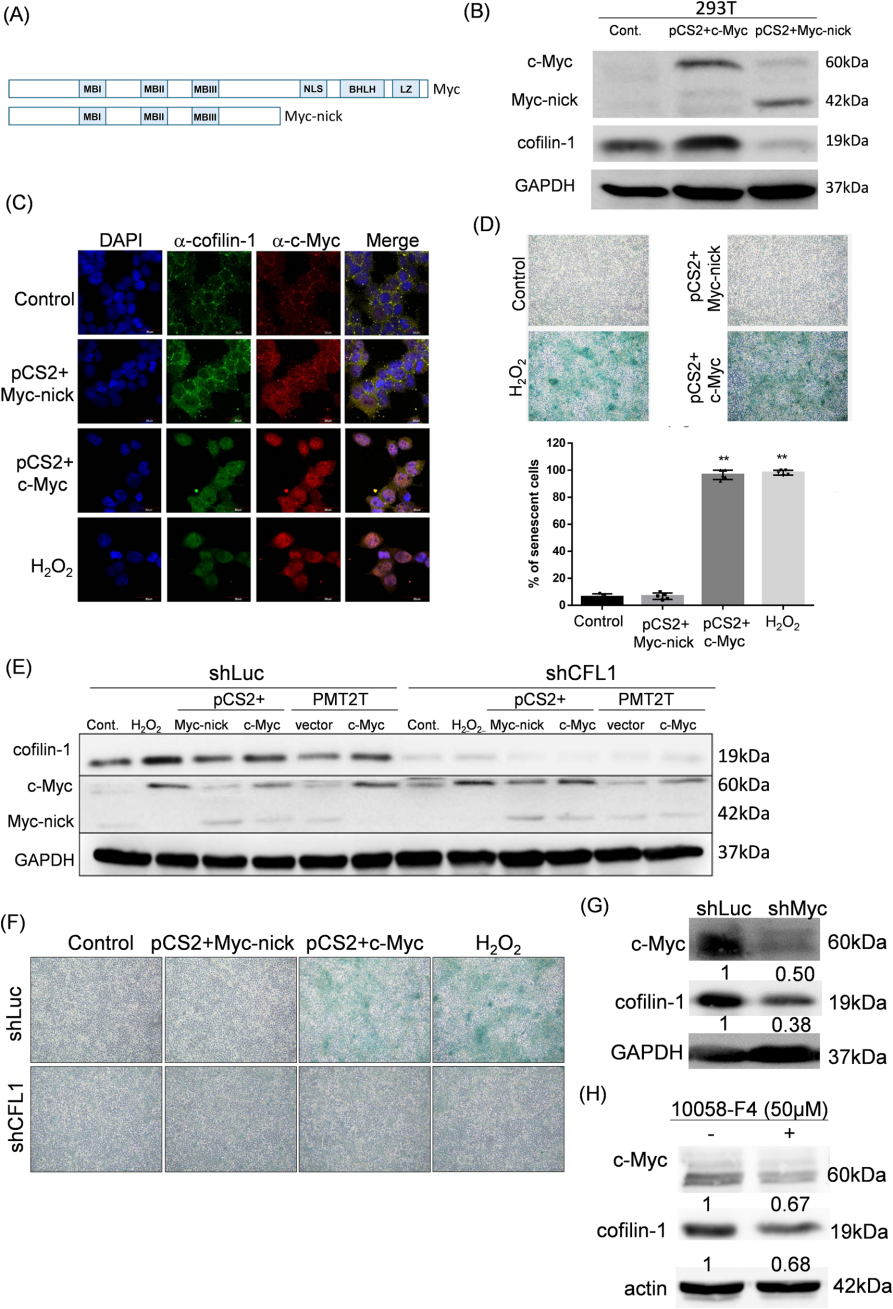
Nuclear entry of c-Myc is required for induction cell senescence and cofilin-1. **A** Schematic representation of full-length c-Myc and truncated Myc-nick; **B** ectopic expression of c-Myc and Myc-nick on the expression of cofilin-1 in 293T cells; **C** visualization of c-Myc, Myc-nick, and cofilin-1 distribution in cells using the immunofluorescence microscopy. H_2_O_2_ treatment was used for comparison; **D** comparison of c-Myc, Myc-nick, and H_2_O_2_ on induction of cell senescence; **E** western blot analysis of cofilin-1 knockdown in cells transduced with c-Myc or Myc-nick using shRNA; **F** effects of cofilin-1 silencing on c-Myc induced senescence; **G** effects of c-Myc knockdown on cofilin-1 expression in H1299 cells transduced with c-Myc shRNA; **H** effects of 10058-F4 on the expression of c-Myc and cofilin-1 after 48 h of treatment. Bands were quantified using the densitometry and normalized to the internal controls. **: *p* < 0.01.

To demonstrate the importance of c-Myc transactivity on cofilin-1 expression and biological response, a truncated version of c-Myc named Myc-nick was recruited [42]. The diagram of Myc-nick primary structure was shown, and domains of nuclear localization signal (NLS), basic helix-loop-helix (bHLH) and leucine zipper (LZ) were deleted from the c-Myc cDNA (Fig. 3A). We next showed that transfection of c-Myc into 293T cells could induce the expression of *CFL1* gene, whereas Myc-nick failed to induce the expression of cofilin-1 (Fig. 3B). The confocal microscopy also demonstrated that Myc-nick could not enter nuclei after transfection compared to c-Myc, and H_2_O_2_ also induced nuclear localization of endogenous c-Myc (Fig. 3C). Furthermore, cells transfected with Myc-nick construct failed to increase SA-β-gal staining compared to cells transfected with c-Myc or treated with H_2_O_2_ (Fig. 3D). We also showed that knockdown of cofilin-1 by shRNA in c-Myc transfected cells and H_2_O_2_ treated cells eliminated the staining of SA-β-gal, although the Myc-nick transfected cells were not affected (Fig. 3E, F). Furthermore, knockdown of c-Myc using shRNA caused decrease of cofilin-1 expression (Fig. 3G). The c-Myc inhibitor 10058-F4 also inhibit the expression of cofilin-1 (Fig. 3H). Taken together, current data suggest that the transcription activity of c-Myc is involved in the expression of *CFL1* gene and cMIS.

### c-Myc exhibits distinct binding affinity to E-box elements on cofilin-1 gene promoter

**Fig. 4:**
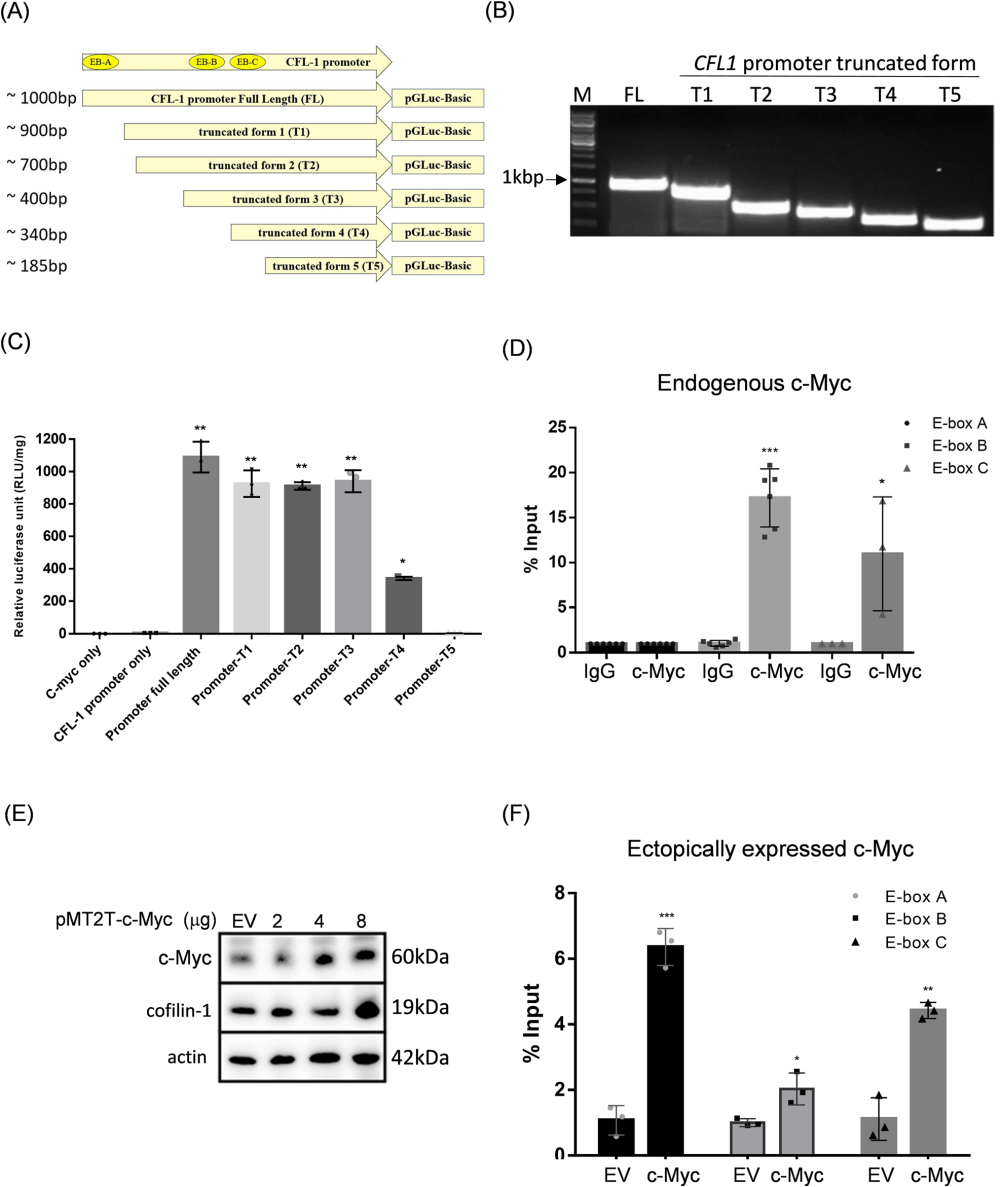
Identification of E-boxes bound by c-Myc in the *CFL1* promoter. **A** Schematic representation of various length of *CFL1* promoter including different amounts of predicted E-boxes; **B** the agarose gel electrophoresis assay for visualization of full-length (FL) and truncated versions of *CFL1* promoter; **C** the luciferase reporter gene assay for comparison of *CFL1* promoter activities at different lengths; **D** the ChIP-qPCR assay for determining the binding ability of endogenous c-Myc onto distinct E-boxes in the *CFL1* promoter; **E** and **F** ectopic expression of c-Myc on binding of E-boxes in the *CFL1* promoter using the ChIP-qPCR assay. EV empty vector. *: *p* < 0.05; **: *p* < 0.01; ***: *p* < 0.001.

To better understand the contribution of three E-box elements on the transactivation of *CFL-1* gene, we used the deletion assay to construct different lengths of *CFL1* gene promoter with different number of E-box elements. The different lengths of *CFL1* gene promoters were designed and generated using the PCR method and they were fused to Gluc reporter gene (Fig. 4A). The PCR products of different lengths of *CFL1* gene promoter were validated using the agarose gel electrophoresis before cloning into the pGluc-Basic vector (Fig. 4B). The luciferase assay showed that full length of *CFL1* gene promoter generated highest luciferase activity, but removal of distal E-box in T1, T2 and T3 constructs barely reduced the luciferase activity (Fig. 4C). However, the luciferase activity was dramatically reduced using the T4 construct, and that was completely diminished using the T5 construct (Fig. 4C). To confirm the physical interaction between c-Myc transcription factor and E-box elements on *CFL1* gene promoter, the ChIP-qPCR assay was introduced to separately detect the binding of c-Myc to these elements using 293T cells. The result showed that endogenous c-Myc primarily bound to the middle E-box (E-Box B) and proximal E-box (E-Box C) of the *CFL1* gene promoter (Fig. 4D). We also co-transfected the c-Myc expressive plasmid and the *CFL1* promoter-GLuc reporter construct into 293T cells for ChIP-qPCR assay. The optimal amount of c-Myc expressive plasmid for induction of endogenous cofilin-1 was determined using the immunoblotting assay (Fig. 4E). Interestingly, transduction of c-Myc could bind to all E-boxes of *CFL1* gene promoter, although the levels were different (Fig. 4F). Current data demonstrate that endogenous c-Myc can bind to the E-boxes closer to the transcriptional initiation site of *CFL1* gene. Over-expression of c-Myc would further bind to all E-boxes on the *CFL1* gene promoter for induction of cofilin-1 expression.

### Cofilin-1 mediated the bystander effect of cMIS

**Fig. 5:**
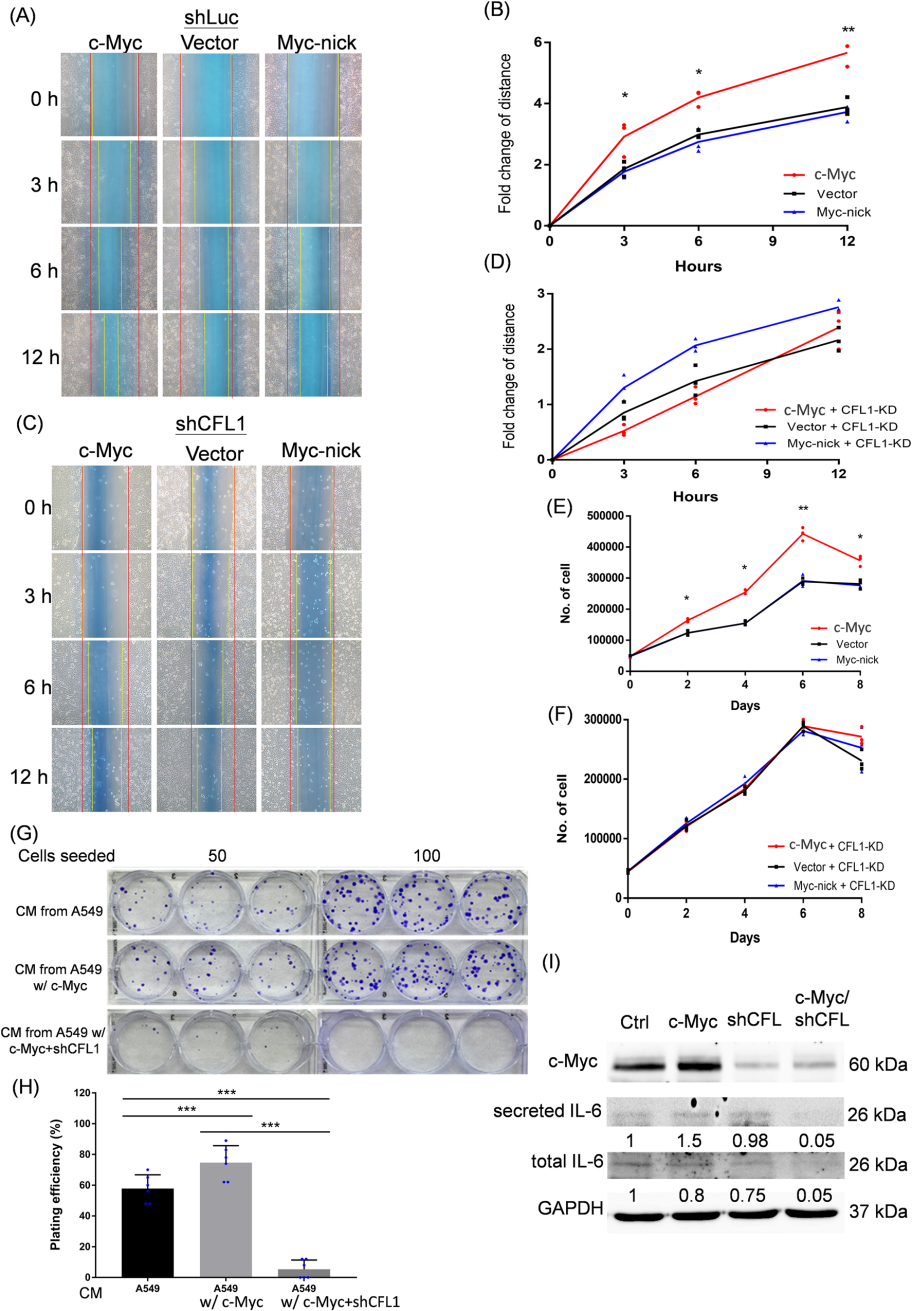
Effects of cofilin-1 on cellular migration and proliferation affected by the CM of c-Myc transduced cells. **A** Wound healing assay for comparing the CMs from c-Myc and Myc-nick transduced A549 cells affecting the migration of untransfected A549 cells; **B** CM from c-Myc transduced cells showed the strongest effect on promoting the migration of untransfected A549 cells; (**C**) and **D** knockdown of cofilin-1 influenced the CM of c-Myc transduced cells on enhancement of cell migration; **E** and **F** hemocytometric analysis with trypan blue staining for evaluating the cell proliferation rate affected by the CM of c-Myc transduced cells with or without knockdown of cofilin-1, respectively; **G** effects of different sources of CM on cells assessed by the colony formation assay; **H** calculation of plating efficiency of CM treated cells by counting the colonies formed in (**G**); **I** western blot analysis of IL-6 in CM and lysates of cells transduced with c-Myc, shCFL1 and combined treatments. IL-6 expression were quantified using the densitometry and normalized to the internal controls. *: *p* < 0.05; **: *p* < 0.01. ***: *p* < 0.001.

It has been reported that senescence associated secretomes may influence the growth and motility of other cells [43, 44]. As cells transfected with c-Myc showed senescent phenotypes, we next explored whether the conditioned media of c-Myc transfected cells would also influence the migration and proliferation of untreated cells. The result showed that the CM of c-Myc transfected A549 cells but not that of Myc-nick transfected one could accelerate the migratory rate of normal A549 cells using the wound healing assay (Fig. 5A, B). Interestingly, knockdown of cofilin-1 in c-Myc transfected cells reduced the cell migratory rate of A549 cells promoted by that CM (Fig. 5C, D). Additionally, the CM of c-Myc transfected cells increased the cell proliferation rate of A549 cells compared to that of vector or Myc-Nick transfected cells (Fig. 5E). Knockdown of cofilin-1 in c-Myc transfected cells significantly decreased the cell proliferative rates promoted by the CM of c-Myc transfected cells (Fig. 5F). Knockdown of cofilin-1 in vector or Myc-Nick transfected cells, on the other hand, did not influence the cell proliferation using the corresponding conditioned media (Fig. 5F). A similar phenomenon was also found in H1299 cells (Supplementary Fig. 4). Additionally, a colony formation assay was used to complement the observation. The results showed that CM from c-Myc transfected cells increased the colony formation in untransfected cells, while CM from c-Myc transfected cells with knockdown of cofilin-1 dramatically reduced the colony number (Fig. 5G, H). To further identify the paracrine factors responsible for the CM-mediated bystander effect, we examined interleukin-6 (IL-6), a key component of the senescence-associated secretory phenotype (SASP). Transfection of c-Myc showed induction of secreted IL-6 in CM, and knockdown of cofilin-1 in c-Myc-transfected cells reduced the level of IL-6 in CM (Fig. 5I). These data suggest that cofilin-1 is important for mediating the c-Myc induced senescence and potent bystander effect.

### Effects of oxidative stress on c-Myc/cofilin-1 regulatory pathway

**Fig. 6:**
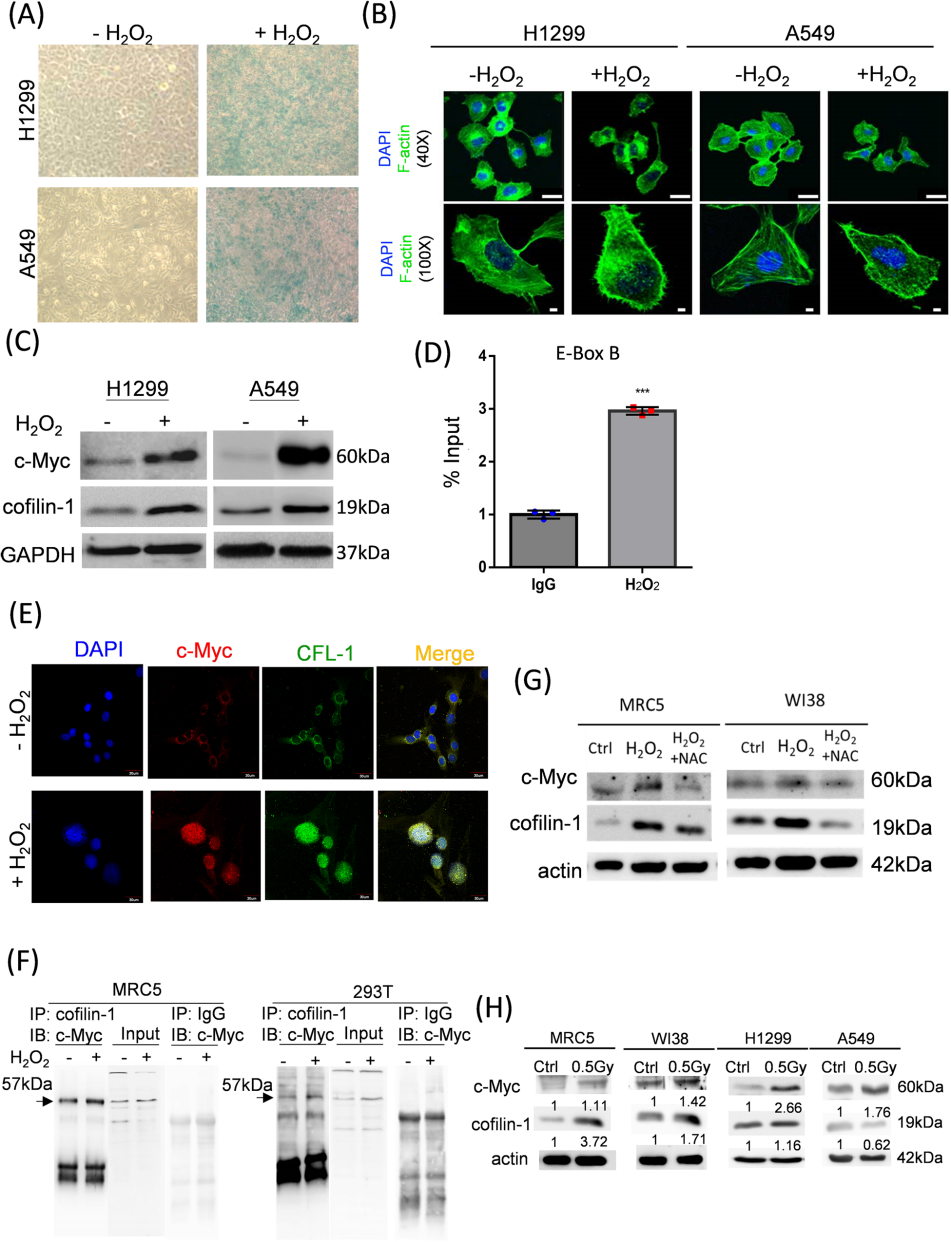
The responses of c-Myc and cofilin-1 to the treatment of H_2_O_2_. **A** SA-β-gal staining for A549 cells and H1299 cells treated with H_2_O_2_ (200 μM); **B** actin cytoskeletal staining using Alexa Fluor™ 488 conjugated phalloidin. Cells were visualized using the confocal microscope at ×40 and ×100 magnificence; **C** western blot analysis for detection of c-Myc and cofilin-1 before and after the treatment of H_2_O_2_; **D** ChIP-qPCR analysis of the binding of c-Myc to the promoter of *CFL1* gene after the treatment of H_2_O_2_; **E** detection of c-Myc and cofilin-1 in MRC-5 cells before and after H_2_O_2_ treatment (20 μM) using the immunofluorescent microscopy; **F** co-immunoprecipitation analysis for detection of the interaction in c-Myc and cofilin-1; **G** expression of c-Myc and cofilin-1 in cells treated with H₂O₂ or co-treated with NAC (5 mM) for 48 h; **H** effects of X-rays on the expression of c-Myc and cofilin-1. ***: *p* < 0.001.

As cells treated with H_2_O_2_ have shown to induce c-Myc and cofilin-1 (Fig. 2C), we further examined the role of oxidative stress played in this regulatory pathway. H_2_O_2_ treatment could induce senescence in A549 and H1299 NSCLC cells (Fig. 6A). It also induced senescence in diploid lung fibroblasts (Supplementary Fig. 5). The organization of stress fibers was also destabilized by H_2_O_2_ using fluorescein conjugated phalloidin staining (Fig. 6B). Again, H_2_O_2_ concomitantly induced c-Myc and cofilin-1 proteins as shown above (Fig. 6C). Interestingly, ChIP-qPCR showed that H_2_O_2_ only enhanced the binding of c-Myc to the middle E-box (designated as E-Box B), while binding to another two E-boxes were undetectable (Fig. 6D and supplementary fig. 6). Using the immunofluorescent microscopy, it was visualized that in MRC-5 cells, c-Myc and cofilin-1 were not only co-upregulated but also co-localized before and after the treatment of H_2_O_2_ (Fig. 6E). Additionally, the co-immunoprecipitation analysis showed that cofilin-1 interacted with c-Myc, and H_2_O_2_ treatment could increase this interaction (Fig. 6F). Furthermore, the antioxidant N-acetylcysteine (NAC) could inhibit H_2_O_2_ induced c-Myc and cofilin-1. (Fig. 6G). In addition to H_2_O_2_, we also examine if X-rays induced oxidative stress would also showed similar effects. The results showed that radiation could induce the expression of both c-Myc and cofilin-1 in different cell types, except A549 cells that cofilin-1 was not co-upregulated with c-Myc (Fig. 6H). In general, oxidative stress would influence the regulation between c-Myc and cofilin-1.

### The association of c-Myc and cofilin-1 expression on survival rates of lung cancer patients

**Fig. 7:**
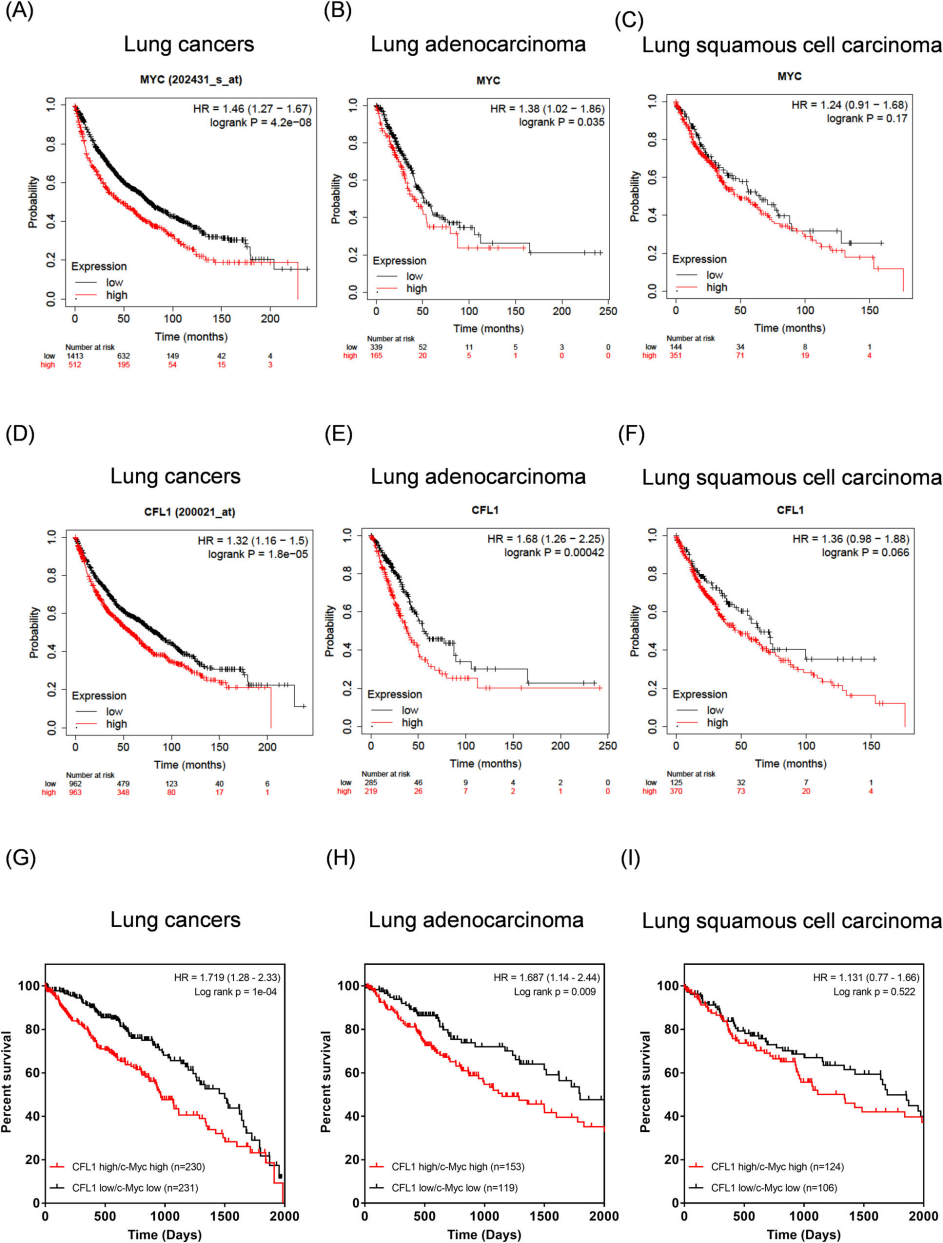
Effects of c-Myc and cofilin-1 on the survival rates of NSCLC patients. **A**–**C** High and low expression of c-Myc on the survival rates of pan-lung cancer, LUAD, and LUSC patients, respectively; **D**–**F** high and low expression of cofilin-1 on the survival rates of pan-lung cancer, LUAD, and LUSC patients, respectively; **G**–**I** combined high and low expression of c-Myc and cofilin-1 on the survival rates of pan-lung cancer, LUAD, and LUSC patients, respectively. *P* < 0.05 was regarded significant.

We next recruited the TCGA database to analyze the expressive level of *c-Myc* gene on patients’ survival. The results showed that high expression of *c-Myc* leads to a poor survival rate in pan lung cancers (Fig. 7A). We next analyzed the expression of *c-Myc* on the survival rates of lung adenocarcinomas (LUAD) and lung squamous cell carcinomas (LUSC), two main histological types of non-small cell lung cancer (NSCLC) that accounts for 85% of all lung cancers [45]. The results showed that high expression of *c-Myc* significantly reduces the survivals of LUAD (Fig. 7B). On the contrary, it barely influences the survival of LUSC (Fig. 7C). We also analyzed the expression of *CFL1* on the survivals of lung cancer patients, and the results were consistent with the influence of *c-Myc* on lung cancers (Fig. 7D–F). Moreover, high expression of both *c-Myc* and *CFL1* significantly decreases the survival rates of LUAD compared to low expression of these genes (Fig. 7G, H), but not that of LUSC (Fig. 7I). Taken together, the regulatory pathway of *c-Myc/CFL1* may influence the survival rates of LUAD subtype of NSCLC.

## Discussion

Despite senescence is caused by limited cell proliferation, aging is ironically the highest risk factor of several majority human cancers [46]. Hence, cell senescence and OIS have been regarded a double-edged sword for cancer progression and treatment [3, 47]. For the tumor suppressive mechanism, OIS mediated by c-Myc over-expression has been reported to be related to the p14ARF-p53 pathway, including a transactivation of ubiquitin-specific protease 10 (USP10) for deubiquitination and stabilization of p14ARF [4, 24]. For the adverse effect of OIS or senescent cells, SASP related inflammatory molecules remain to be the primary factor for promotion of cancer development [48–50]. The underlying mechanism, from OIS to SASP mediated paracrine effects on malignancy is obscure. In this study, we demonstrated that c-Myc induce senescence is accompanied by its nuclear entry and transactivation of *CFL1* gene, which is required for the bystander effect mediated by c-Myc. This regulatory axis would interpret the harmful effect of c-Myc induced senescence, at least in part. Compared to Ras, the first oncogene identified in OIS, and BRAF or AKT oncogene, c-Myc is a transcription factor that directly binds to the promoters of various genes. Exploring whether cofilin-1 serves as the mediator of SASP induced solely by c-Myc or also by other forms of OIS would be intriguing.

OIS is known an unstable phenomenon that can be escaped by various mechanisms, including derepression of the hTERT locus [7], downregulation of histone demethylases [51], stemness-associated reprogramming [48], reorganization of topologically associated domains (TADs) [52] and epigenetic changes [53]. We have also found that a longer time of c-Myc transfection seemed incapable of maintaining G1 phase arrest in WI-38 cells and H1299 cells, but not in A549 cells. It appears that escape of cMIS is cell type dependent, and the potent mechanisms may be interesting to further investigate.

Since Hayflick and his colleagues delineated the limitation of proliferation in normal diploid human lung fibroblasts as replicative senescence (RS), the morphological change accompanied by the cell cycle cessation are recognized as typical characteristics of cellular senescence after numbers of passage in cultured cells [54]. The senescence-associated morphological profiles (SMAPs) used for distinguish the senescent cells from proliferating cells showed that OIS also exhibits strikingly potent profiles [55]. It has been reported that cellular senescence expresses increased organization of cytoskeleton, accumulation of nuclear globular actin, and up-regulation of cofilin-1 [28, 56, 57]. These phenomena could also be detected in cMIS, suggesting that the morphological alterations in RS and OIS share similar mechanisms.

Low dose radiation (LDR) has been reported to induce immediately early response genes including c-Myc [58]. Furthermore, c-Myc is involved in LDR induced senescence [59]. As the primary effect of radiation is to induce oxidative stress [60], we also showed that LDR (0.5 Gy) could induce c-Myc and cofilin-1 in several cell lines used in this study, except A549 cells in which cofilin-1 was not up-regulated with c-Myc (Fig. 6H). As mentioned, cofilin-1 is a potent marker of poor prognosis after radiotherapy [38]. Inhibition of c-Myc during radiotherapy may suppress the adverse effect of LDR induced oxidative stress [59].

Cofilin-1 often exhibits up-regulation in various cancer types via transactivation of the *CFL1* gene. For instance, the transcription factor 7-like 2 (TCF7L2) can directly bind to *CFL1* promoter to promote tumor progression in human bladder cancer [61]. Hypoxia-inducible factor-1α (HIF-1α) has also been found to bind to the promoter of *CFL1* gene in advancing hepatocellular carcinoma [62, 63]. Here we showed the binding of c-Myc onto *CFL1* promoter, and identified the differential interaction of c-Myc on three E-boxes. Notably, the distal E-box showed lowest influence on transactivation of *CFL1* gene, and it seems no other elements can regulate the expression of *CFL1* gene beyond the mid E-box in 293T cells expressing low level of endogenous c-Myc [64]. This result suggests that c-Myc should be essential for basal expression of *CFL1* gene, especially through the mid and proximal E-box on the *CFL1* promoter. On the other hand, over-expression of c-Myc showed strongest binding to the distal E-box followed by the proximal E-box, but weakest binding to the mid E-box. This opposite action of c-Myc binding on E-boxes of *CFL1* gene would partially interpret the oncogenic role of over-expressed c-Myc in tumor development.

Several lines of evidence have shown that the senescence-associated secretory phenotype (SASP) can promote tumor progression by contributing a pro-tumorigenic microenvironment via autocrine and paracrine pathways [43, 47, 48, 65]. SASP is known to promote the malignancy by affecting the proliferation, migration and invasion of non-senescent cancer cells adjacent to senescent tissues [66]. Senescent cells per se are also resistant to apoptosis and become persistent in tissues, which may constitutively secret SASP and generate a chronic inflammatory microenvironment benefit for cancer growth [67]. The pro-inflammatory cytokines IL-6 is known as the primary factor of SASP [68]. We have examined the level of IL-6 in c-Myc transfected cells, and it was increased in the CM. Interestingly, knockdown of cofilin-1 in c-Myc transfected cells could compromise this effect. However, other factors in SASP remain to be investigated in the future. As previously noted, cofilin-1 is accumulated during the progression of senescence, yet its potential influence on the secretion of SASP remains undetermined. Here we have shown that in NSCLC cells, knockdown of cofilin-1 suppresses c-MIS and the bystander effects. It would be interesting to investigate if cofilin-1 can modulate the SASP mediated by c-MIS via specific mechanisms.

Oxidative stress is known as a risk factor of lung carcinogenesis but also promotes cell cycle arrest and apoptosis in advanced lung cancers, causing cancer cell senescence finally [69]. Up-regulation of cofilin-1 by c-Myc mediated transactivation could also be promoted by oxidative stress, although only one of the E-boxes responded to this stimulation. Surprisingly, it appears that c-Myc can physically interact with cofilin-1, and oxidative stress strengthen this effect. Whether the interaction of c-Myc and cofilin-1 is important for the oncogenic ability of c-Myc or for the cofilin-1 mediated cell senescence is intrigue to be investigated in the future.

Clinically, it is not surprisingly that high expression of *c-Myc* and *CFL1* are primarily associated with low survival rates in LUAD subtype of NSCLC patients. LUAD is a predominant subtype of NSCLC because it represents 50–60% of all NSCLC cases [70]. However, similar treatments of LUAD and LUSC with distinct histologic and biological signatures are usually administrated in clinics because of limited comprehensive knowledge on both subtypes [71]. Indeed, several identified biomarkers between LUAD and LUSC showed distinguishable value, and certain biomarkers even display prognostic significance in LUAD rather than LUSC [45]. Compared to independent biomarkers, our data showed the c-Myc and cofilin-1 representing a regulatory axis to affect the survival rate of LUAD. Exploring this molecular event may be intriguing and crucial when devising strategies for precise theranostics.

In summary, current data demonstrated that c-Myc can transactivate *CFL1* gene, which is required for c-Myc induced senescence. In addition, c-Myc could differentially recognize distinct E-boxes in the *CFL1* promoter, but over-expression of c-Myc would bind to all E-boxes to up-regulate cofilin-1. In lung cancer cells, cofilin-1 is essential for cMIS and resultant bystander effect that promote their proliferation and mobility. The c-Myc/cofilin-1 regulatory axis is responsible for the poor survival rate in LUAD subtype of NSCLC, and it may contribute to design of related strategies for lung cancer treatment.

## Materials and methods

### Cell culture

Human non-small cell lung adenocarcinoma A549 cells and H1299 cells, human embryonic kidney 293T cell, and human diploid fibroblasts MRC-5 cell were purchased from m American Type Culture Collection (ATCC) and cultured in Dulbecco’s Modified Eagles Medium (DMEM, Gibco, NY, USA) containing 10% Fetal Bovine Serum (FBS, SAFC Bioscience, Kansas, USA), 1% penicillin-streptomycin (P/S) solution, and maintained at 37 °C in a humid incubator with 5% CO_2_ and 95% air. Human diploid fibroblasts WI-38 cells were cultured in Minimum Essential Medium (MEM, Gibco, NY, USA) containing 10% FBS, 1% P/S solution under the same condition. The passage numbers of MRC-5 cells and WI-38 cells were around 9 and 22, respectively.

### Plasmids

The pMT2T-c-Myc construct and the pMT2T vector were kindly provided by Dr. Muh-Hwa Yang from National Yang Ming Chiao Tung University, Taipei, Taiwan. The pCS2+c-Myc and the pCS2+Myc-nick were generously provided by Dr. Robert N. Eisenman from Division of Basic Sciences, Fred Hutchinson Cancer Research Center. Seattle, USA. The pCFL1p-Gluc reporter gene construct contained a ~1kbp *CFL1* promoter, which was amplified using the polymerase chain reaction (PCR) and fused to pGLuc-BASIC plasmid through EcoRI and BamHI restriction enzyme sites. The pEGFP-N1 plasmid was used to evaluate the transfection efficiency by following the instruction of PolyJet™ In Vitro DNA Transfection Reagent (SignaGen Laboratories, Frederick, MD, USA).

### MYC inhibitor treatments

Cells were treated with the MYC–MAX dimerization inhibitor 10058-F4 (50 μM) for 48 h. Control cells received equivalent volumes of vehicle (DMSO).

### Cell cycle and apoptosis analysis

Cells (1 × 10^6^) were fixed in 70% ice-cold ethanol and kept in 4 °C overnight. Subsequently, cells were centrifuged to remove ethanol and resuspended in 1X phosphate buffered saline (PBS, Biological Industries, Kibbutz Beit Haemek, Israel) supplemented with 0.1 mg/mL RNase A for 45 min. Cells were then collected and treated using 0.1 mg/mL propidium iodide (PI) followed by sieving them through 0.37 μm mesh. The treated cells were subjected to the flow cytometer (Cytomic FC 500, Beckman Coulter, Inc., Fullerton, CA, USA) for further analysis. For apoptosis analysis, the 5X binding buffer (50 mM HEPES, 700 mM NaCl, 12.5 mM CaCl2, pH 7.4) was pre-diluted (1:4) in deionized water before treatment. Cells were then washed with 1X PBS. Cells were gently resuspended in pre-diluted binding buffer, and adjusted to 2–5 ×10^5^/mL. The cell mixtures (195 μL) was added with 5 μL Annexin V-FITC (Sigma-Aldrich, St. Louis, MO, USA) and incubated at room temperature (r.t.) for 10 min. Subsequently, cells were collected and washed with 1X PBS and resuspended in pre-diluted binding buffer containing PI solution (20 μg/mL) for 15 min. Cells were filtered through 0.37 μm mesh followed by flow cytometric analysis.

### Senescence-associated β-galactosidase (SA β-gal) staining assay

Cells (3 ×10^5^) cultured in 60 mm dishes were rinsed with PBS and then treated with the fixing solution (2% formaldehyde and 0.2% glutaraldehyde in 1X PBS) at room temperature for 5 min. The fixing solution was replaced with the freshly prepared staining solution [40 mM citric acid (pH 6.0)/sodium phosphate, 1 mg/mL X-gal, 5 mM potassium ferricyanide and 5 mM potassium ferrocyanide (Sigma-Aldrich, St. Louis, MO, USA), 150 mM NaCl, 2 mM MgCl_2_ in distilled H_2_O], and placed in a 37 °C oven for overnight. The stained cells were visualized and acquired using an inverted phase contrast microscope (CK-40 Olympus Co, Japan) equipped with a digital camera (PowerShot A260, Canon Inc., USA). Senescent cells were counted in 5 ~ 10 random fields (approximately 100–250 cells in total) to determine the percentage of SA-β-gal positive cells.

### Detection of F-actin and G-actin

Alexa Fluor™ 488 phalloidin (ThermoFisher Scientific, Waltham, MA, USA) was used to stain and visualize the cellular actin cytoskeleton according to the manufacture’s instruction. Cells (8 × 10^3^) were seeded and cultured in Nunc™ Lab-Tek™ II Chamber Slide™ System (Thermo Fisher Scientific, Waltham, MA, USA). After 48 h with or without transfection of c-Myc construct, cells were washed twice with 1X PBS and fixed using 4% paraformaldehyde for 10 min. Triton X-100 (0.1%) was then used for cell permeabilization for 5 min, and then rinsed with 1X PBS three times. Alexa Fluor™ 488 phalloidin was added with 4’,6’-diamidino-2-phenylindole (DAPI) for 20-30 min. For detection of G-actin, Alexa Fluor™ 594 DNase I conjugate (ThermoFisher Scientific, Waltham, MA, USA) was used [72]. The chamber slide was then rinsed, mounted using Fluoromount-G™ Mounting Medium (ThermoFisher Scientific, Waltham, MA, USA), sealed with nail polish and subjected for microscopic visualization. The fluorescent cell images were acquired using the inverted laser scanning confocal microscope equipped with a cooled CCD camera (Olympus FV-1000, Becker & Hickl GmbH, Berlin, Germany).

### Western blot analysis and antibodies

Cells were lysed in protein lysis buffer (50 mM Tris-HC, 120 mM NaCl, 0.5% NP-40 and 1 mM PMSF) at 4 °C for 20 min, and the extracts were collected by centrifugation at 4 °C for another 20 min. Thirty μg protein lysates were resolved on sodium dodecyl sulfate-polyacrylamide gel electrophoresis (SDS-PAGE). The separated proteins were electrotransferred to a polyvinylidene difluoride (PVDF) membrane (BioTrace^TM^ NT; Pall, Port Washington, NY, USA). The membrane was immersed with blocking buffer [4% non-fat milk dissolved in Tris-buffered saline buffer (20 mM Tris–HCl, pH 7.4, 3 mM KCl, and 140 mM NaCl) containing 0.1% Tween®20 detergent, TBST] at r.t. for 1 h and replaced with fresh TBST buffer containing primary antibody at 4 °C overnight. Subsequently, the PVDF membrane were washed with TBST buffer at r.t. for 15 min three times, and then incubated with horseradish peroxidase (HRP)-conjugated secondary antibody at r.t. for 1.5 h. The membrane was then exposed using Western Lighting *Plus*-ECL (Perkin-Elmer Inc., Waltham, MA, USA), and the specific luminescent signals were detected using the Fujifilm LAS-4000 Luminescence/Fluorescence imaging system (Fujifilm Co., Tokyo, Japan) and quantified using the ImageJ software (ImageJ ver 1.47), (National Institutes of Health, Bethesda, MD, USA). The anti-vasodilator-stimulated phosphoprotein (VASP), anti-Ezrin/Radixin/Moesin (ERM), and anti-phospho-ERM antibodies of the actin reorganization antibody sampler kit (Cell Signaling Technology, Danvers, MA, USA) were used. In addition, other primary antibodies used included anti-cofilin-1 (GTX102156), anti-profilin-2 (GTX108589), anti-ADF/Destrin (GTX114170), anti-c-Myc (GTX109636), anti-IL-6 (GTX110527), anti-actin (GTX114170, Genetex Inc., Irvine, CA, USA), anti-p27^kip1^ (610241, BD Transduction Laboratories, Franklin Lakes, NJ, USA), anti-IL-6 (GTX110527), and anti-glyceraldehyde-3-phosphate dehydrogenase (GAPDH) (G8795, Sigma-Aldrich Inc., St. Louis, MO, USA). The secondary anti-rabbit or anti-mouse antibody was purchased from Sigma-Aldrich Inc.

### Reverse-transcriptase-quantitative polymerase chain reaction (RT-qPCR)

Total RNA was isolated using the Trizol reagent (Life-Technologies Co, Grand Island, NY, USA). The NanoDrop ND-1000 (Thermo Fisher Scientific, Waltham, MA, USA) was used to quantify extracted RNA. SuperScript^TM^ II RT Kit (Thermo Fisher Scientific, Waltham, MA, USA) was used for synthesis of first strand cDNA according to the product guideline. In brief, 250 ng random primer, 10 mM dNTP, and 1 μg total RNA was mixed in a nuclease-free tube. The mixture was heated at 65 °C for 5 min and quickly placed on ice. Subsequently, 5X First-Strand Buffer, 0.1 M DTT, and 200 units of SuperScript^TM^ II RT were added into the mixture at r.t. for 10 min, followed by incubation at 42 °C for 50 min. The reaction was inactivated at 70°C for 15 min. The forward primer for detection of cofilin-1 transcript was 5′ – CGAGTCTGCGCCCCTTAA – 3′, and the reverse primer was 5′ – GCAGTTTGCTTGCAATTCATG – 3′. The forward primer for U6 transcript internal control was 5′ – CGCTTCGGCAGCACATATAC – 3′, and the reverse primer was 5′ – TTCACGAATTTGCGTGTCAT – 3′. The cDNA products obtained above were mixed with the Fast SYBR Green Master Mix (Applied Biosystems, Life-Technologies Co, Grand Island, NY, USA) and amplified in the StepOne Plus Real-Time PCR System (Applied Biosystems, Life-Technologies Co, Grand Island, NY, USA) following the manufacturer’s instructions. Each datum represented the mean of four repeats.

### Luciferase reporter assay

Cells (3 × 10^5^) were co-transfected the *CFL1* promoter (full-length or different promoter deleted version) harboring reporter construct and c-Myc expressive plasmid into cells using PolyJet™ In Vitro DNA Transfection Reagent (SignaGen Laboratories, Frederick, MD, USA). After transfection for 48 h, cells were washed with 1X PBS and lyzed in the passive lysis buffer (Promega Corporation, Madison, WI, USA) and placed at –80 °C for 2 h. Subsequently, the cell lysates were collected and mixed with the reporter assay buffer (50 mM Glycylglycin, 1 M MgSO_4_, 10 mg/ml of BSA, and 0.5 M EDTA) mixed with 100 mM ATP (Sigma-Aldrich, St. Louis, MO, USA), 1 M dithiothreitol, and 50 mM D-Luciferin (Xenogen, Challenger Dr, Alameda, CA, USA). The luminescence signals were detected using a Mutilabel Counter (Perkin Elmer Wallac 1420 victor^2^, Waltham, MA, USA). The unit of bioluminescent signals was count per second (CPS) and normalized to the concentrations of cell lysates.

### Chromatin immunoprecipitation—qPCR (ChIP-qPCR) assay

The ChIP assay was conducted following the manufacturer’s instructions for the ChIP assay kit (Millipore, Billerica, MA, USA). Briefly, approximately 1 × 10^7^ cells were treated with 1% formaldehyde (Sigma-Aldrich, USA) at 37 °C for 10 min to crosslink proteins to DNA, then washed three times with cold PBS containing a 1% protease inhibitor cocktail (Calbiochem, San Diego, CA, USA). The lysate was then sonicated with an Ultrasonic Disruptor S-4000 (MISONIX, USA) to shear the chromatin into fragments ranging from 200 to 1000 bp, as verified by electrophoresis. The pre-cleared chromatin fragments were incubated with Protein A agarose beads and a specific anti-c-Myc antibody (Cell Signaling Technology, Danvers, MA, USA), or an anti-rabbit IgG1 antibody (Genetex Inc., Irvine, CA, USA) as a negative control, at 4 °C overnight. Following extensive washing, the bound DNA fragments were eluted after reversing the crosslinks with proteinase K digestion, with the reaction stopped by heating at 65 °C for 4 h. The eluted DNA was analyzed using a QuantStudio™ 3 Real-Time PCR System (Thermo Fisher Scientific, USA) with specific primers. The following primer sets were used; for distal E-box: forward: 5′ – GGCGGCTAGCCTCATATTCATG – 3′; reverse: 5′ – TGGGGGCCCTAATCCTATAAA – 3; for middle E-box: 5′ – CCCTGTGCCTTTTCCTCCTAT – 3′; reverse: 5′ – TGGAATCCCGACCTAAAGCA – 3; for proximal E-box: forward: 5′ – ATGCAAAACCCTAACCTCACTCA – 3′; reverse: 5′ – AGGCGGCGCTCAGTAAAAT – 3.

### Immunofluorescence staining

Cell fixation and permeabilization were executed described as above. The slides were blocked using the immunofluorescence blocking buffer containing goat serum as a protein blocker (Cat#12411, Cell Signaling Technology, Danvers, MA, USA) at 37 °C for 45 min. The primary antibody, including anti-cofilin-1 (GTX102156) and anti-c-Myc (GTX20032, Genetex Inc., Irvine, CA, USA) were then used to treat cells at 37 °C for 1.5 h. Subsequently, the slides were rinsed with pre-warmed washing buffer in jar at 45 °C for 10 min three times on a shaker. Cells were then incubated with secondary antibodies (FITC-conjugated anti-rabbit IgG #F0382 and TRITC conjugated anti-mouse IgG #T7782) and DAPI at 37 °C for 45 min. After several times of rinse in 1X PBS, the slide was mounted, sealed, and visualized using the same confocal microscope as mentioned above.

### Lentivirus-based vector for gene knock-down system

293T cells were seeded in a 60 mm dish, and co-transfected with 2.5 μg pLKO.1-shMyc, pLKO.1-shCFL1 or pLKO.1-shLuc (control) plasmid, and packaging plasmids (0.25 μg pMD.G and 2.25 μg pCMV-ΔR8.91) purchased from RNAi core facility at Sinica Academy, Taipei, Taiwan. After transfection for 48 h, the culture medium was replaced with DMEM containing 10 mg /mL bovine serum albumin (BSA). Subsequently, the medium was collected twice after 12 and 24 h. The virus soup was centrifuged at 110,000 × *g* at 4 °C for 2 h, and collected pellets were resuspended in serum-free medium. The concentrated virus soup was used for infection by adding 8 μg/ml polybrene (Sigma-Aldrich, St. Louis, MO, USA) into the culture medium for 24 h of treatment. The fresh medium was then replaced and kept for another 24 h prior to further analysis.

### CM preparation for wound healing and cell proliferation assay assay

Cells were seeded for transfection of different plasmids for 48 h. Subsequently, the CM were harvested from the media of cultured cells by centrifuged at 1.5 × 10^3^ rpm at r.t. for 5 min. The composition of the CM, including FBS was not changed after collection. The CM was added to untransfected cells for analysis of their proliferation and migration rate. Cell migration rate was determined using the Wound healing assay. Confluent cells were in 60 mm dishes were straightly scratched using pipette tip for triplicate repeats. The migration of cells was visualized under the bright field microscope and the images were acquired using the equipped digital camera at 3, 6 and 12 h after initial scratch. The migration distance of cells was quantified using the ImageJ software as mentioned before. For cell proliferation, 3 × 10^4^ cells seeded in a 12-well plate and cultured in a 37 °C humid incubator for 8 day. Cells were trypsinized and counted using the hemocytometry after staining with trypan blue solution (Thermo Fisher Scientific, Waltham, MA, USA). For colony formation assay, 50 and 100 cells were seeded in 6-cm culture dishes after they were treated with different sources of CM. The plates were stained by crystal violate in 70% ethanol after incubation for 14 days. The colonies were counted and the plating efficiency was calculated by the formula: (formed colonies/cells seeded) x 100%.

### Co-immunoprecipitation analysis

Cells grown on monolayer were lyzed in lysis buffer containing 2% protease inhibitor cocktail set III (Calbiochem, San Diego, CA). One out of tenth total protein was reserved for input. Equal amount of cell extracts (~200 μg) collected from different treatments were incubated with anti-cofilin-1 antibody for 2 h at 4 °C with gentle shaking, and then mixed with Protein-A/G agarose (Santa Cruz Inc., Santa Cruz CA) overnight. Immune complexes were centrifuged at 1.3 × 10^4 ^rpm for 20 min, and the pellets were added with pre-clean buffer (10% BSA in lysis buffer) at 4 °C for 30 min. Subsequently, the lysates were washed three times by 1X PBS and centrifuged at 1.3 × 10^4^ rpm for 3 min each round. The pellets were resuspended in 5 μl SDS-protein loading dye and ran on a 12% polyacrylamide gel and detected using anti-c-Myc antibody as mentioned in Western blot analysis.

### Ionizing radiation treatment

Cells were exposed to X-rays irradiation at a dose of 0.5 Gy using an X-rays irradiator (X-RAD 225, Precision X-Ray, North Branford, CT, USA) at a dose rate of 1.34 Gy/min. Cells were harvested at 1 h post-irradiation for protein analysis.

### In silico database analysis

The gene expression and overall survival using publicly available datasets were downloaded from Kaplan-Meier Plotter (https://kmplot.com/analysis/). For assessing the clinical information and genomic matrix file of The Cancer Genome Atlas (TCGA) database, the Kaplan-Meier method with the log-rank test was employed to analyze the correlation of *CFL1* and *c-Myc* with the survival rates of lung cancer, lung adenocarcinoma, and lung squamous cell carcinoma cohorts using the USCS Xena browser website (https://xenabrowser.net/) with public datasets, respectively.

### Statistical analysis

The Student’s *t* test and one-way analysis of variance (ANOVA) were used to determine the significance of statistical results compared to the control. The p-value < 0.05 was regarded significant difference. The software used for statistical analysis was GraphPad Prism V.9.0 (GraphPad Software, Boston, MA, USA).

## Supplementary information

Supplementary Figure 1

Supplementary Figure 2

Supplementary Figure 3

Supplementary Figure 4

Supplementary Figure 5

Supplementary Figure 6

Supplementary Figure 7

Supplementary Figure 8

Supplementary Figure 9

Supplementary Figure 10

Supplementary Figure 11

Supplementary Figure 12

Supplementary Figure 13

Supplementary Figure 14

Supplementary Figure 15

Supplementary Figure 16

Supplementary Figure 17
