# Supplementary figures and images for: c-Myc transactivates CFL1 to induce senescence-like phenotype and potentiate the bystander effects for the migration and proliferation in lung cancer cells
Source: Cell Death Discov. 2026 Mar 26;12:192. doi: 10.1038/s41420-026-03065-3 (PMC13144380; doi:10.1038/s41420-026-03065-3)

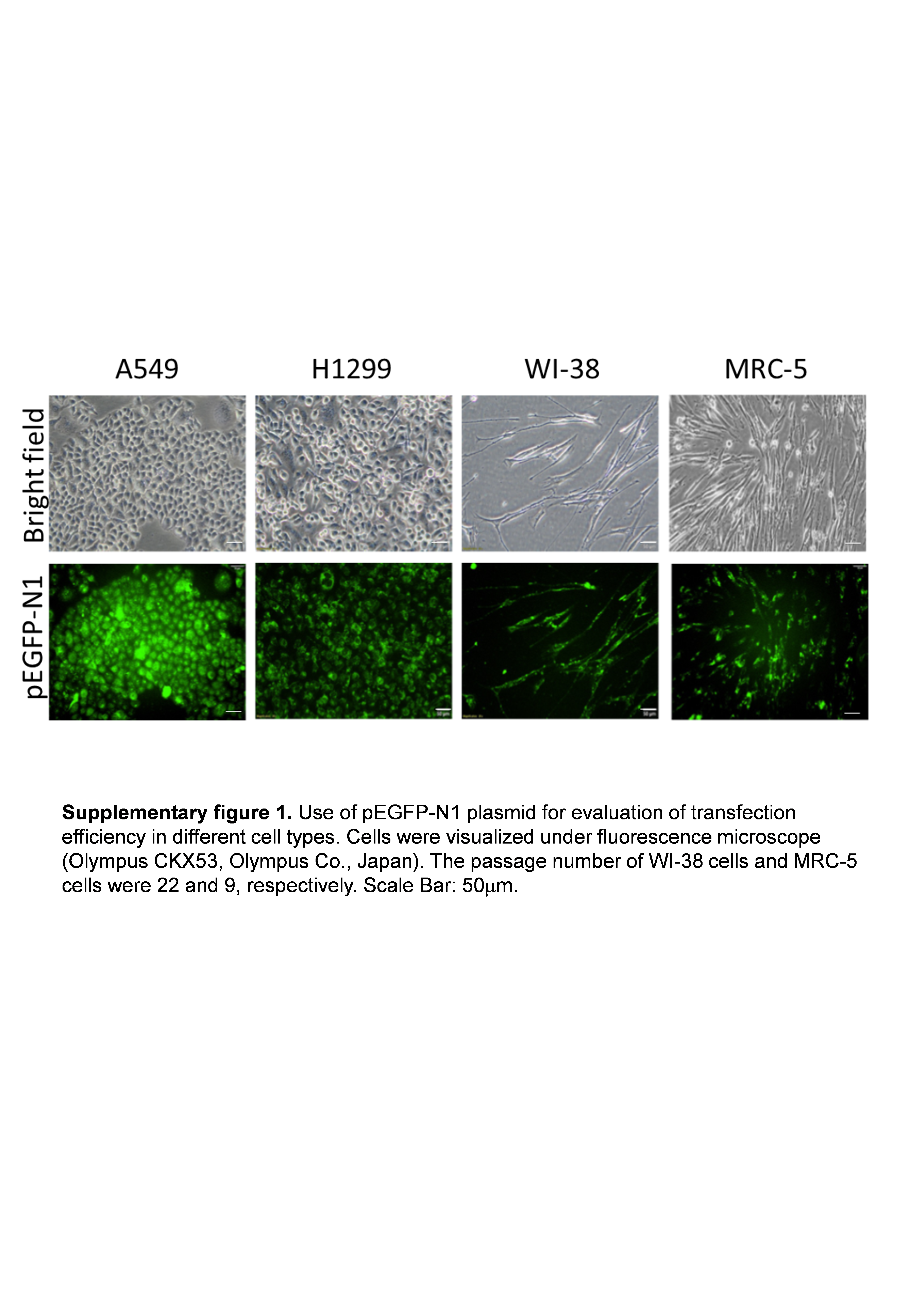

Supplement: Supplementary file 1 — Supplementary Figure 1 [file 41420_2026_3065_MOESM1_ESM.tif]

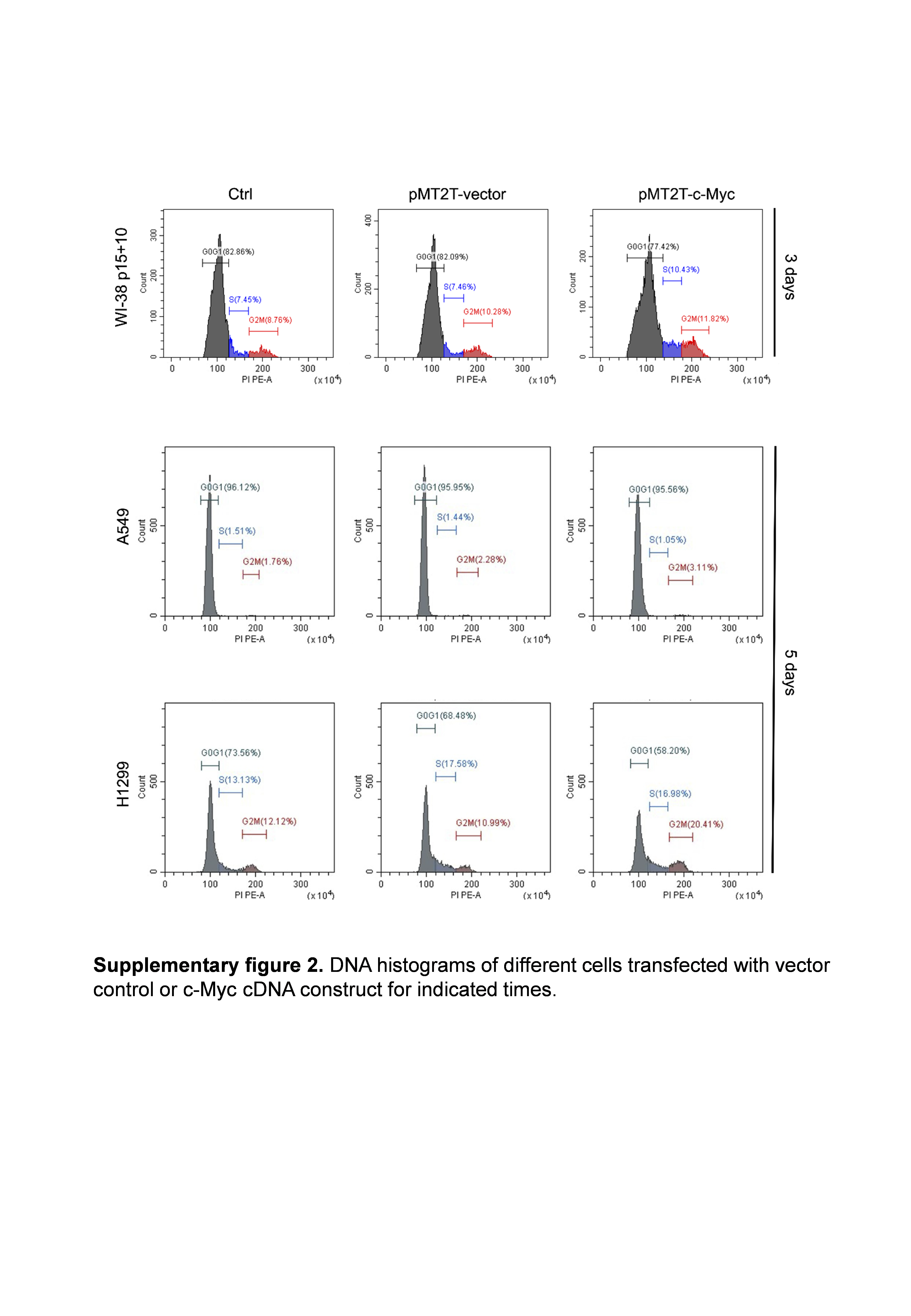

Supplement: Supplementary file 2 — Supplementary Figure 2 [file 41420_2026_3065_MOESM2_ESM.tif]

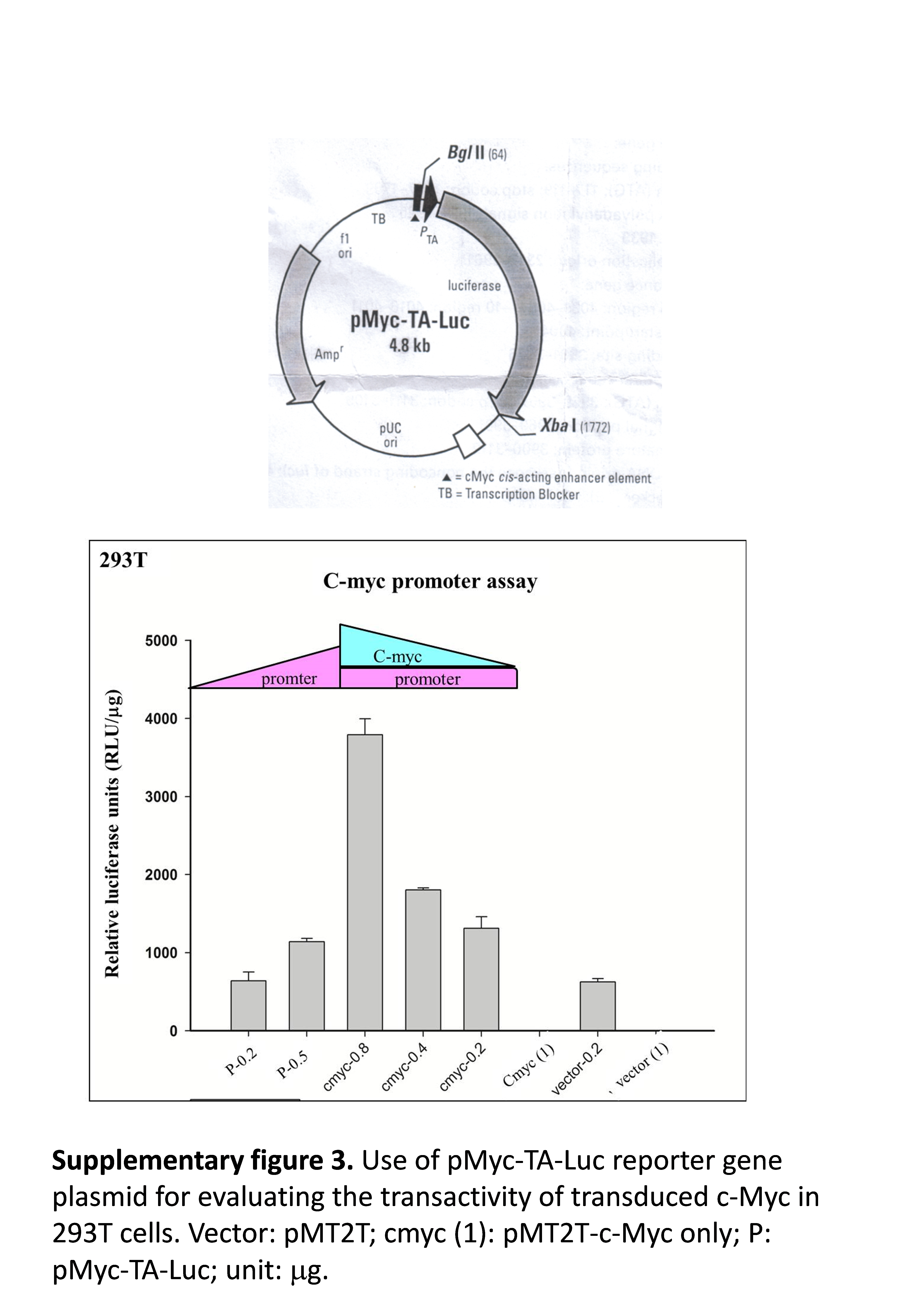

Supplement: Supplementary file 3 — Supplementary Figure 3 [file 41420_2026_3065_MOESM3_ESM.tif]

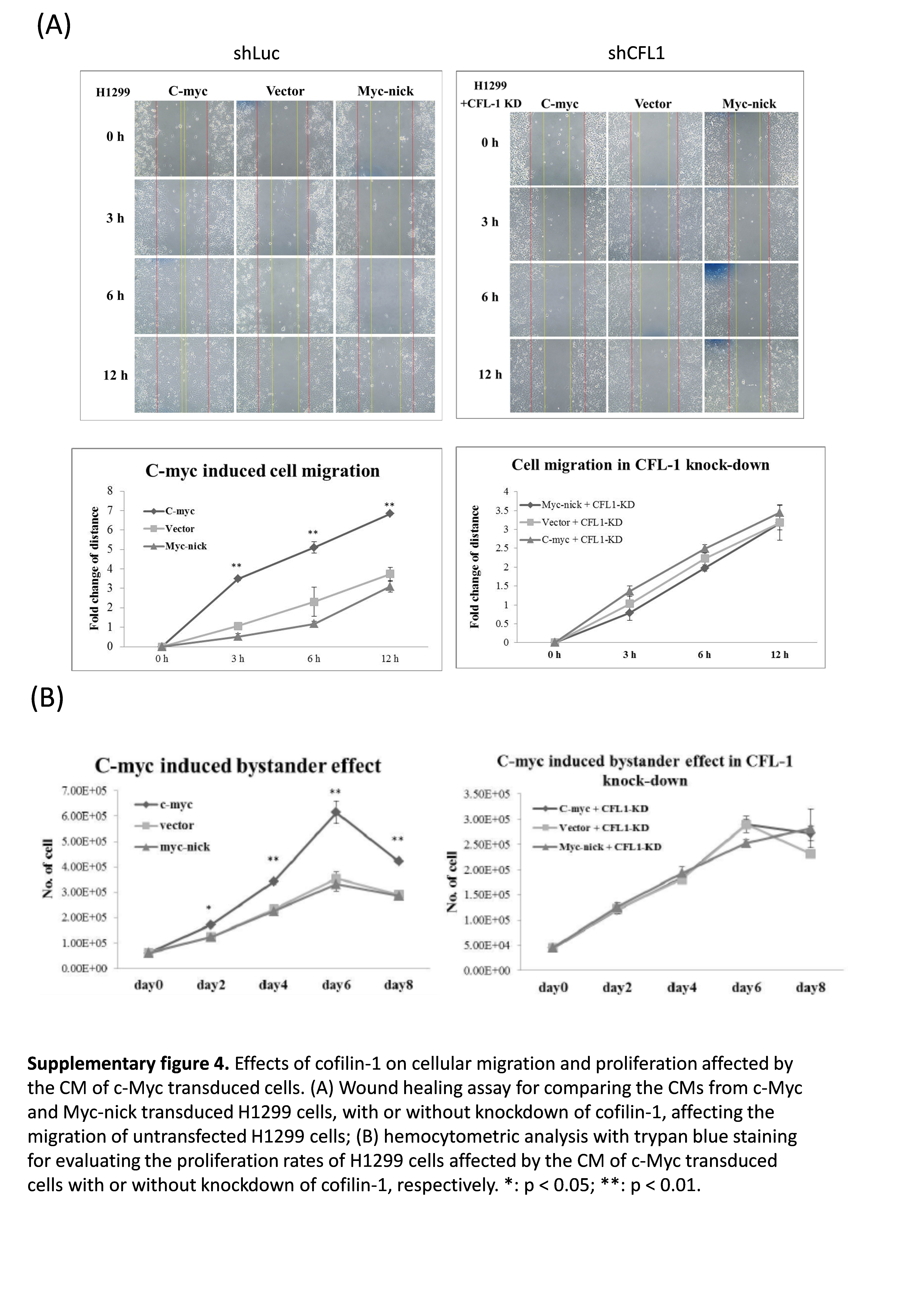

Supplement: Supplementary file 4 — Supplementary Figure 4 [file 41420_2026_3065_MOESM4_ESM.tif]

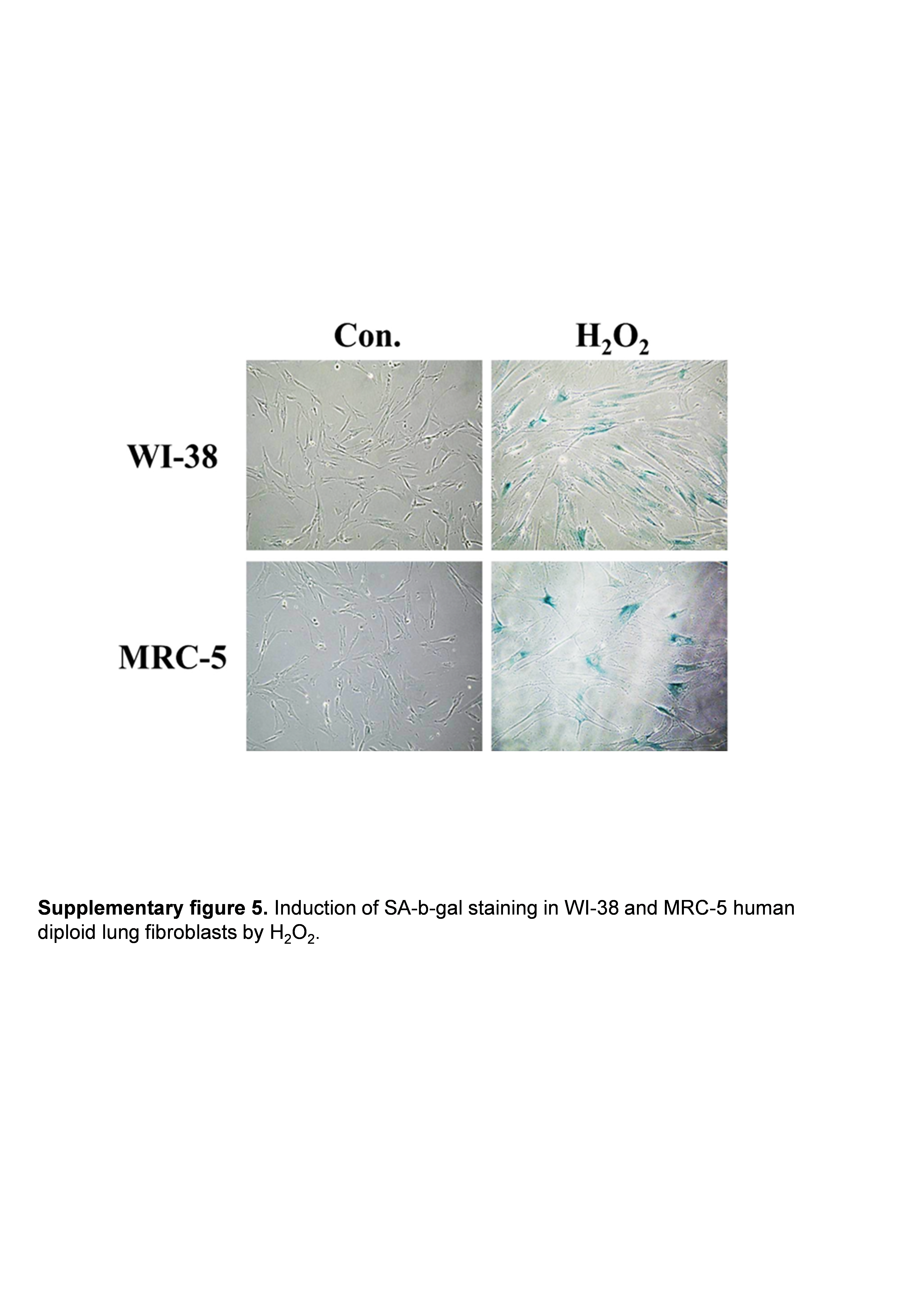

Supplement: Supplementary file 5 — Supplementary Figure 5 [file 41420_2026_3065_MOESM5_ESM.tif]

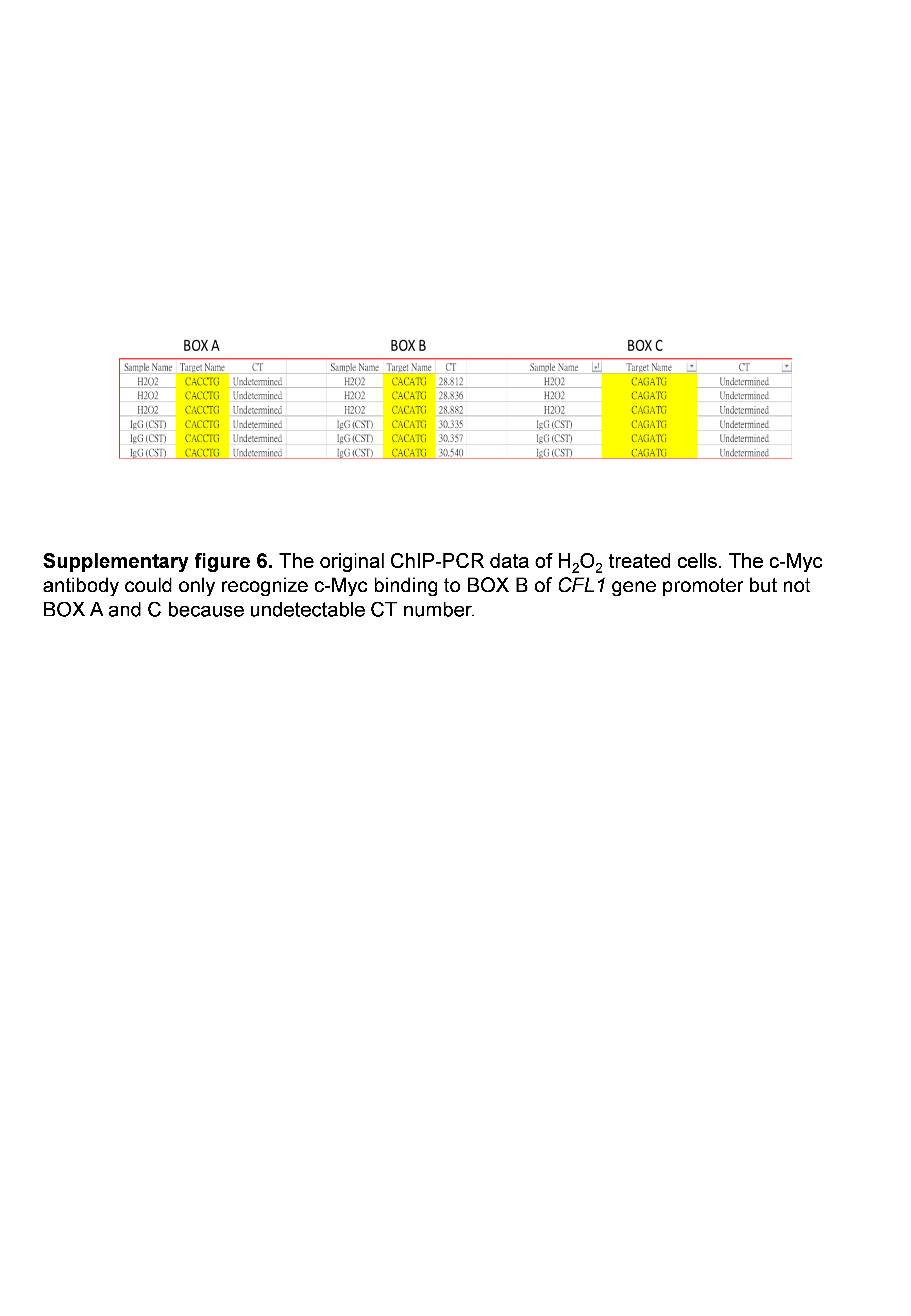

Supplement: Supplementary file 6 — Supplementary Figure 6 [file 41420_2026_3065_MOESM6_ESM.tif]

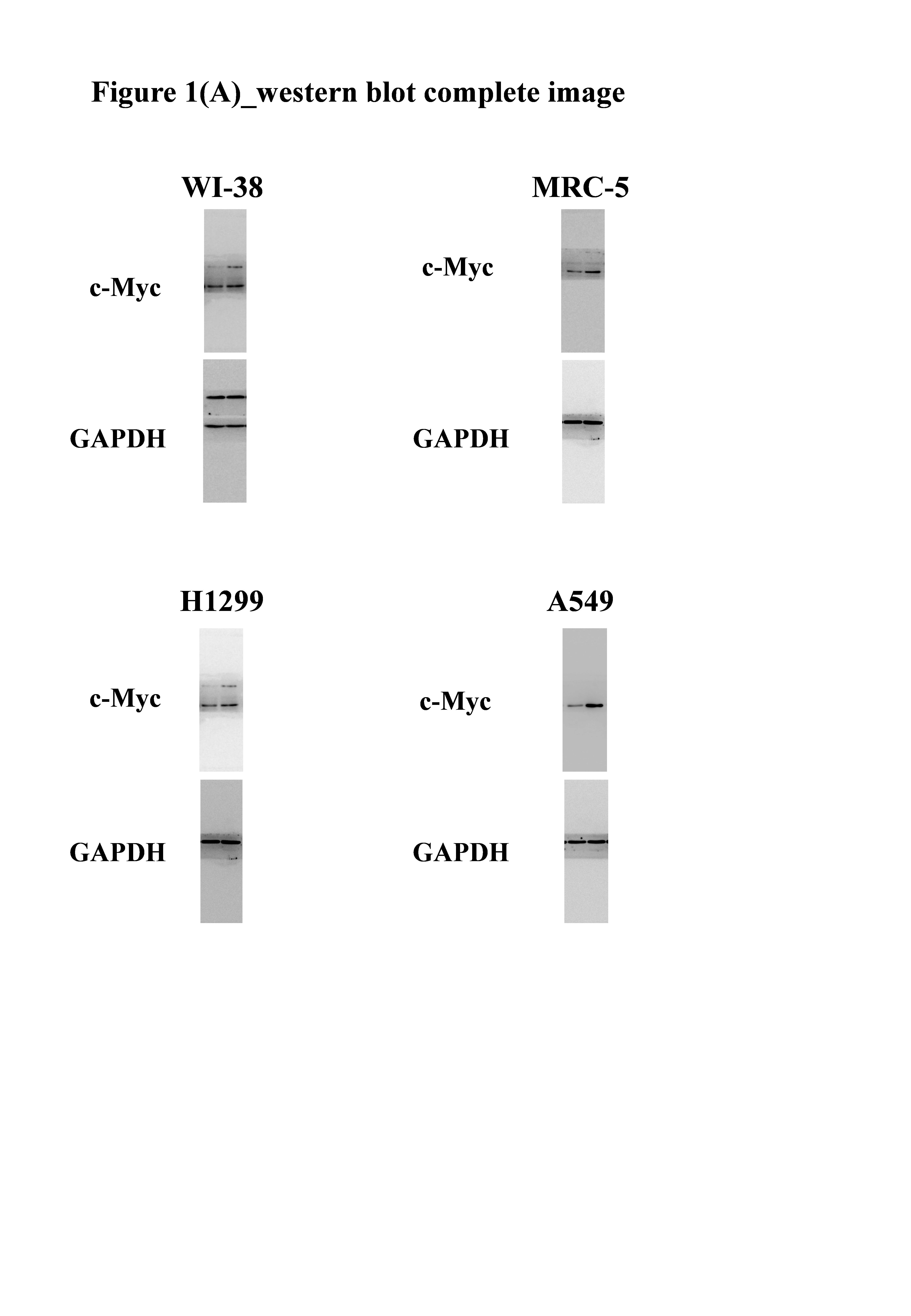

Supplement: Supplementary file 7 — Supplementary Figure 7 [file 41420_2026_3065_MOESM7_ESM.tif]

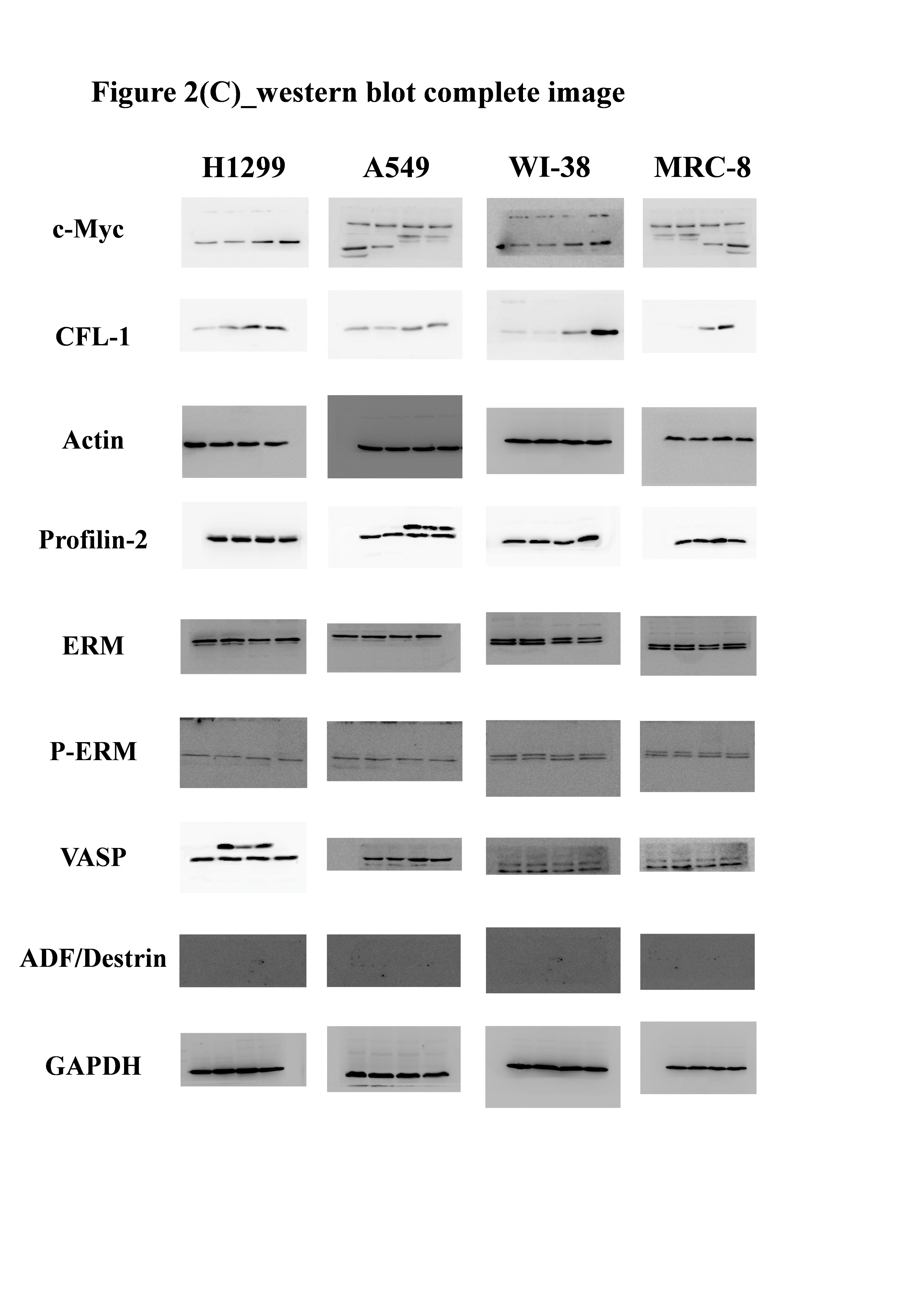

Supplement: Supplementary file 8 — Supplementary Figure 8 [file 41420_2026_3065_MOESM8_ESM.tif]

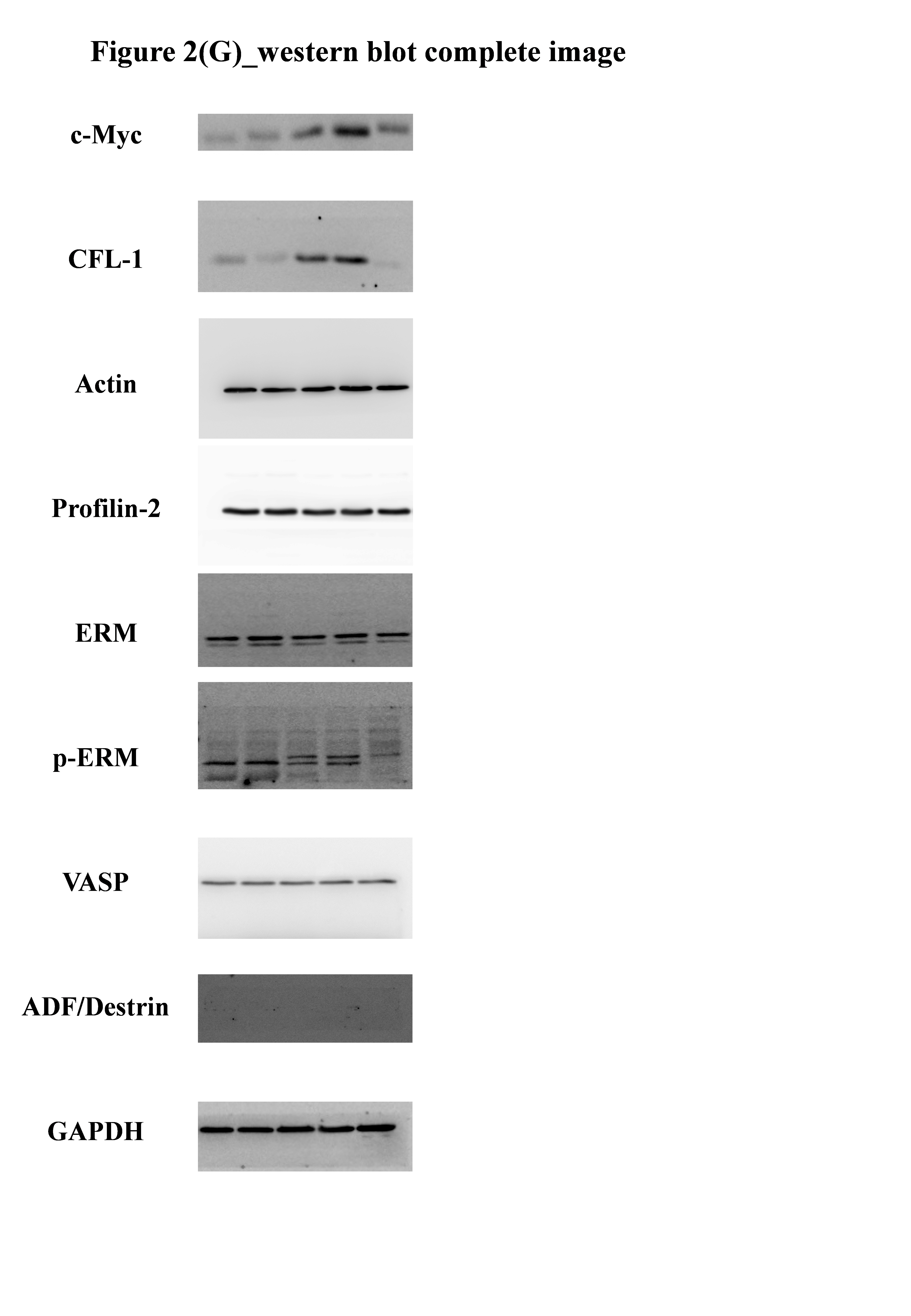

Supplement: Supplementary file 9 — Supplementary Figure 9 [file 41420_2026_3065_MOESM9_ESM.tif]

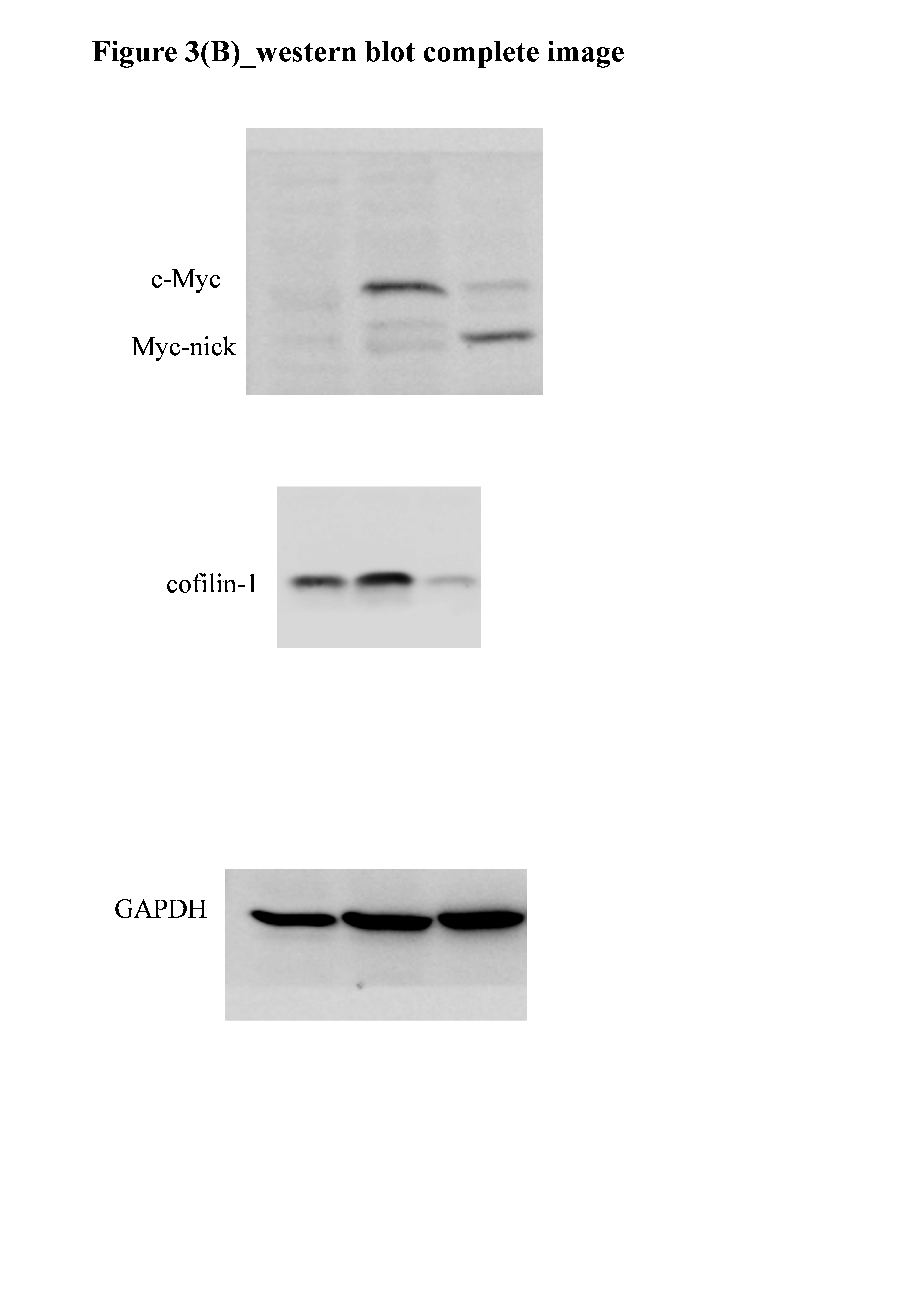

Supplement: Supplementary file 10 — Supplementary Figure 10 [file 41420_2026_3065_MOESM10_ESM.tif]

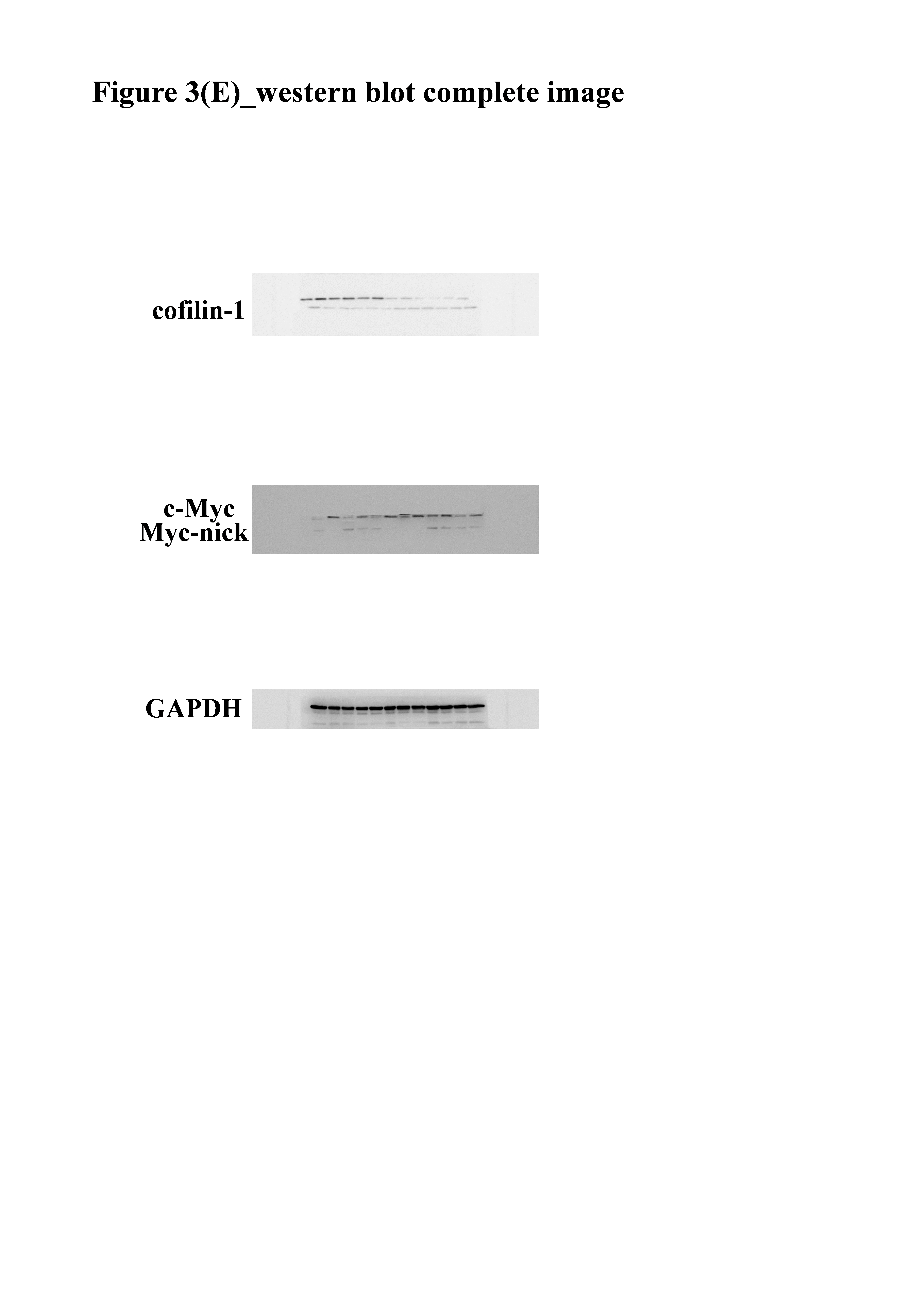

Supplement: Supplementary file 11 — Supplementary Figure 11 [file 41420_2026_3065_MOESM11_ESM.tif]

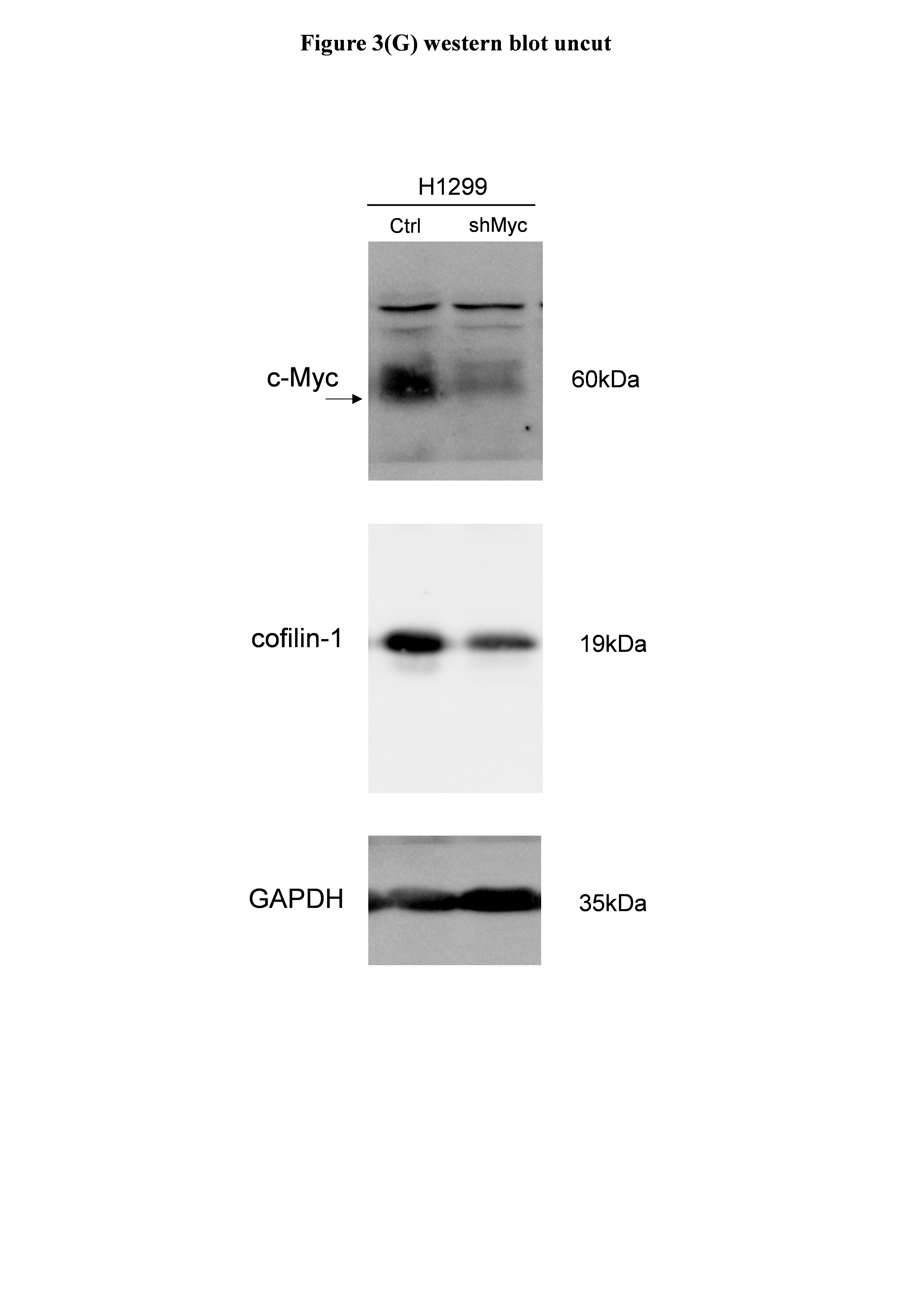

Supplement: Supplementary file 12 — Supplementary Figure 12 [file 41420_2026_3065_MOESM12_ESM.tif]

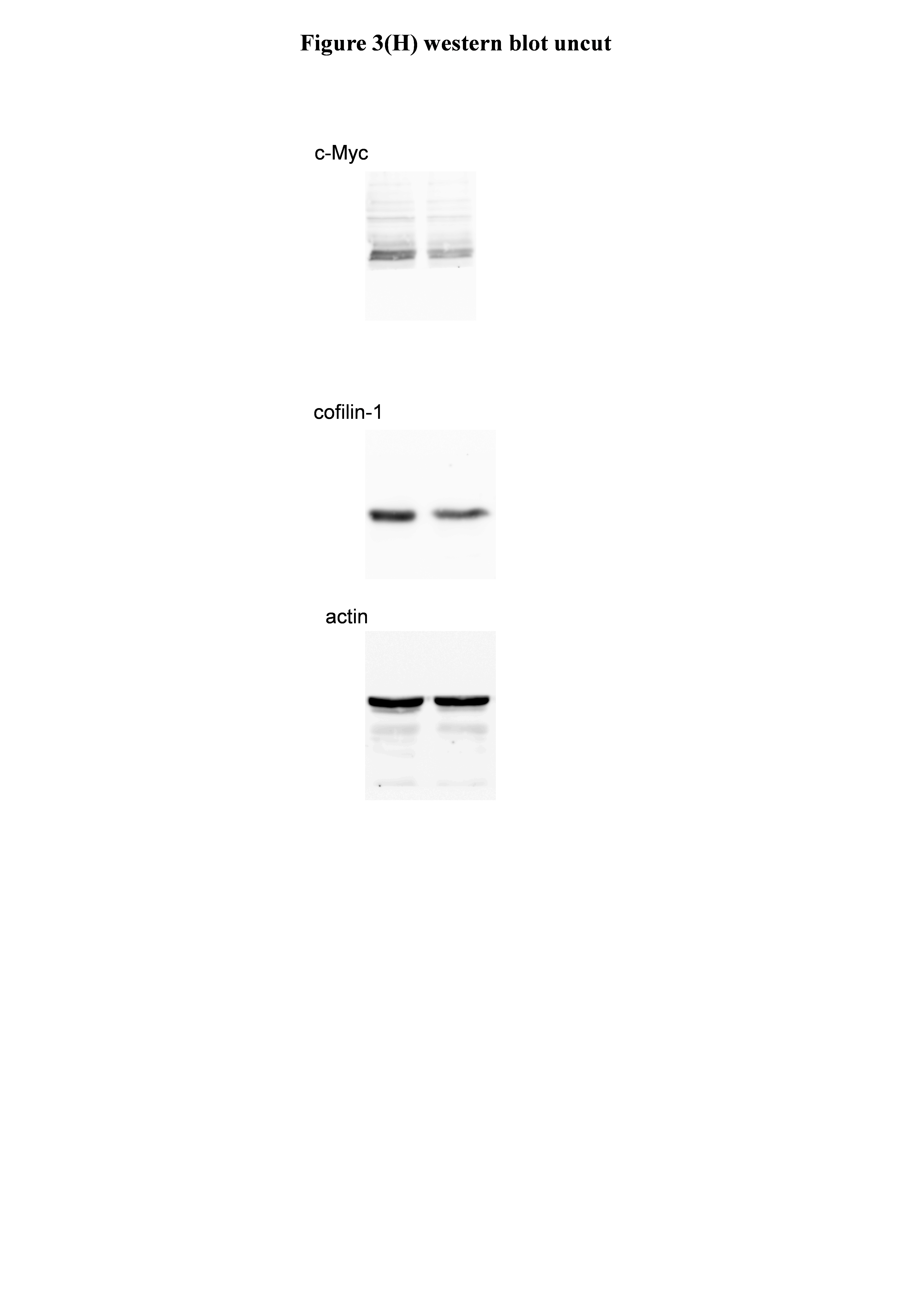

Supplement: Supplementary file 13 — Supplementary Figure 13 [file 41420_2026_3065_MOESM13_ESM.tif]

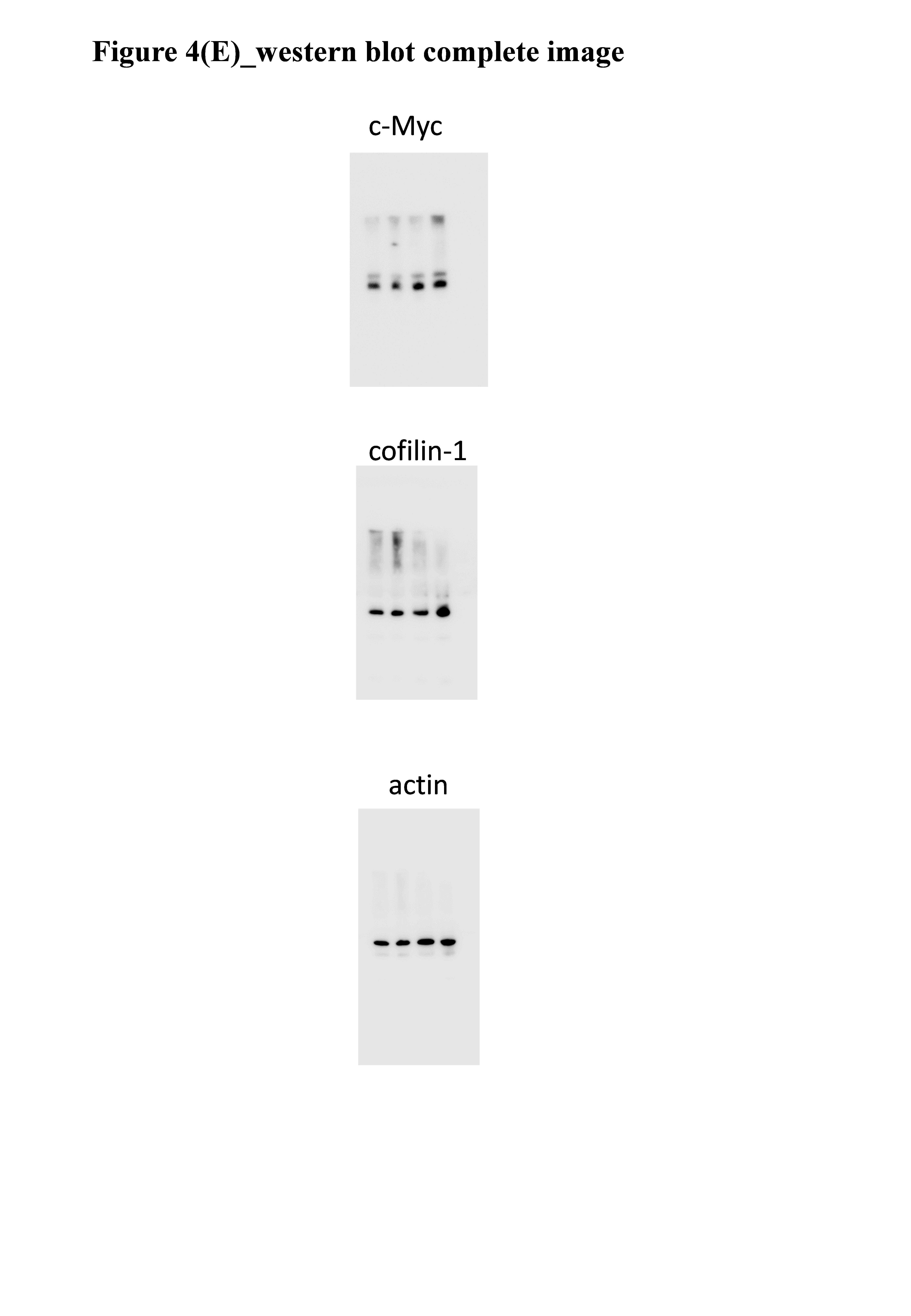

Supplement: Supplementary file 14 — Supplementary Figure 14 [file 41420_2026_3065_MOESM14_ESM.tif]

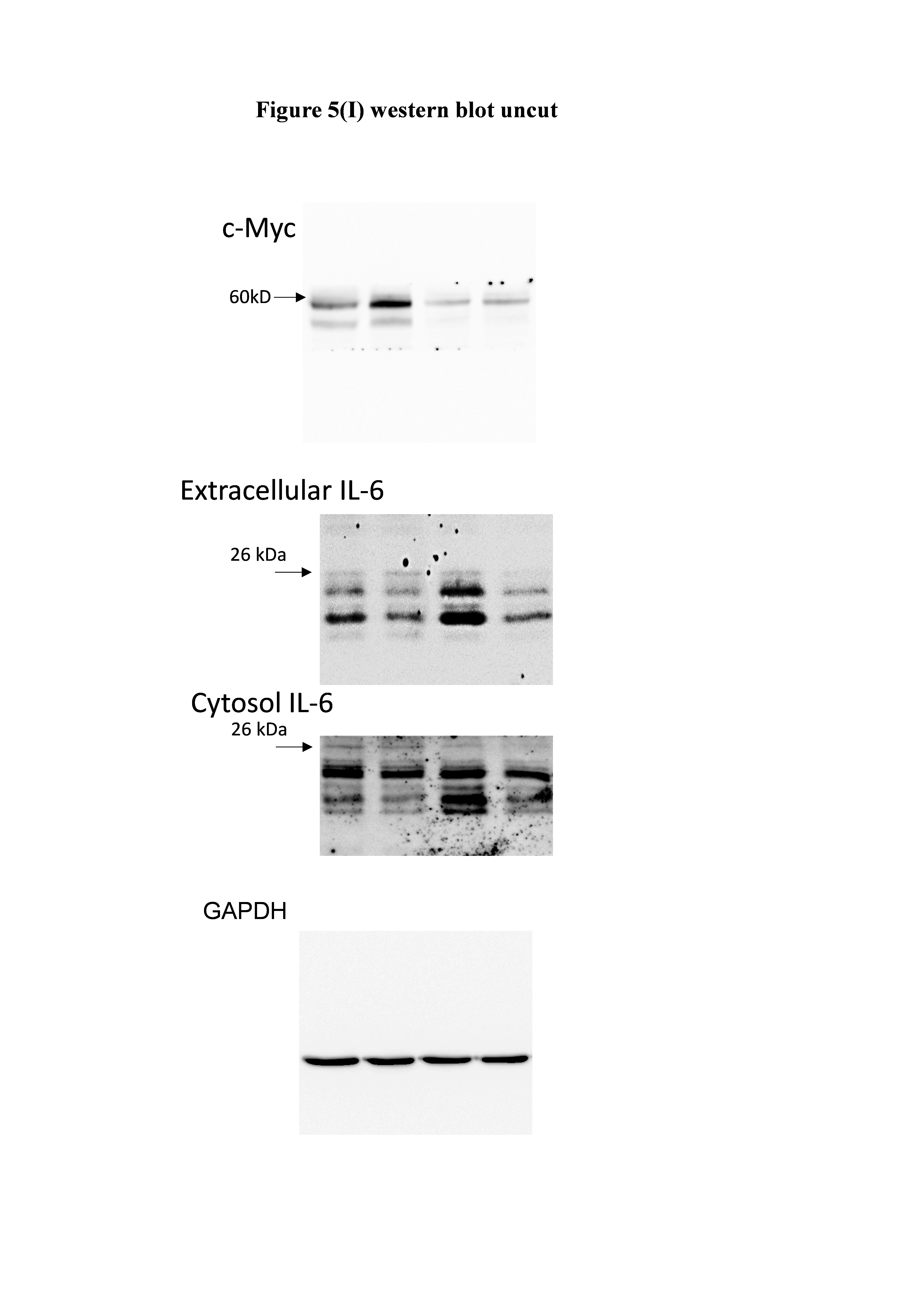

Supplement: Supplementary file 15 — Supplementary Figure 15 [file 41420_2026_3065_MOESM15_ESM.tif]

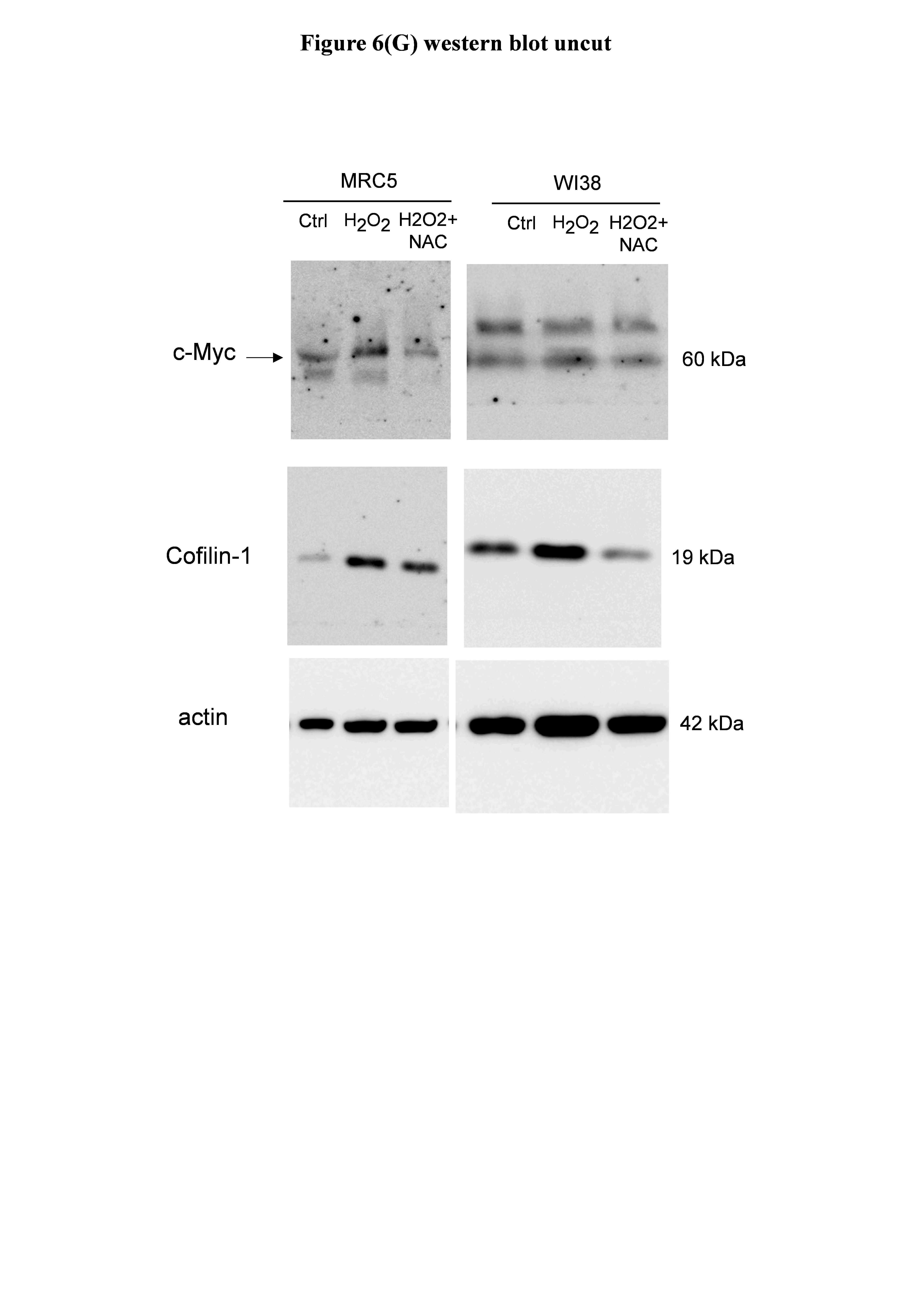

Supplement: Supplementary file 16 — Supplementary Figure 16 [file 41420_2026_3065_MOESM16_ESM.tif]

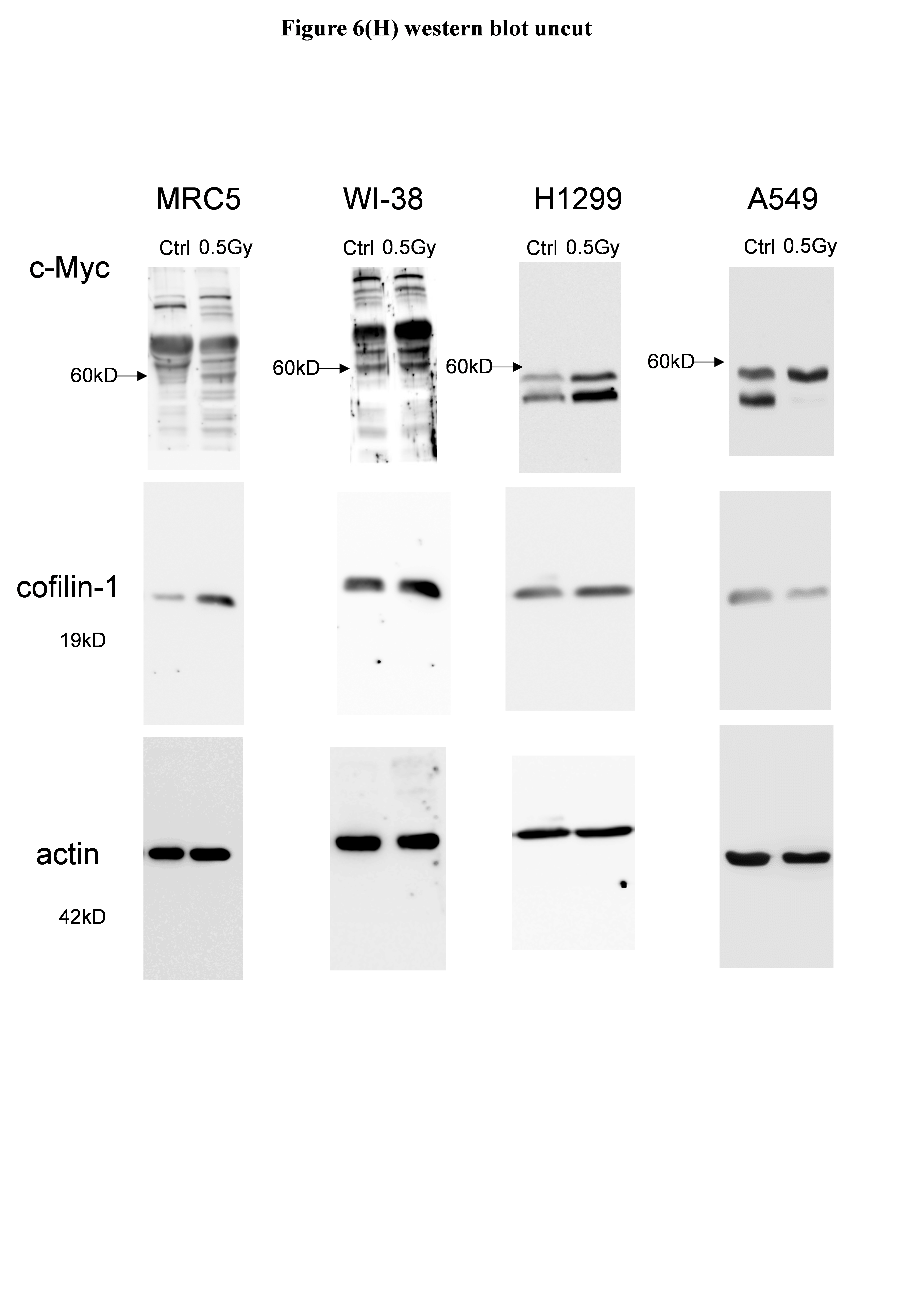

Supplement: Supplementary file 17 — Supplementary Figure 17 [file 41420_2026_3065_MOESM17_ESM.tif]
